# Octanoic acid mitigates busulfan-induced blood-testis barrier damage by alleviating oxidative stress and autophagy

**DOI:** 10.1186/s12944-024-02157-2

**Published:** 2024-06-11

**Authors:** Chun Cao, Hong Zhang, Zhaowanyue He, Kemei Zhang, Zhang Qian, Jiaming Shen, Lu Zheng, Mengqi Xue, Shanshan Sun, Chuwei Li, Wei Zhao, Jun Jing, Rujun Ma, Xie Ge, Bing Yao

**Affiliations:** 1 Department of Reproductive Medicine, Affiliated Jinling Hospital, The First School of Clinical Medicine, Southern Medical University, 305 Zhongshan East Road, Nanjing, 210002 China; 2 Center of Reproductive Medicine, Jinling Hospital, Affiliated Hospital of Medical School, Nanjing University, 305 Zhongshan East Road, Nanjing, 210002 Jiangsu China; 3 Reproductive Medical Center, Jinling Hospital Department, Nanjing Medical University, Nanjing, 210002 Jiangsu China; 4 College of Life Sciences, Nanjing Normal University, Nanjing, 210023 Jiangsu China

**Keywords:** Medium-chain fatty acids, Octanoic acid, Busulfan, Spermatogenesis disorder, Blood-testis barrier, Autophagy, Oxidative stress, Male infertility

## Abstract

**Background:**

The management of male infertility continues to encounter an array of challenges and constraints, necessitating an in-depth exploration of novel therapeutic targets to enhance its efficacy. As an eight-carbon medium-chain fatty acid, octanoic acid (OCA) shows promise for improving health, yet its impact on spermatogenesis remains inadequately researched.

**Methods:**

Mass spectrometry was performed to determine the fatty acid content and screen for a pivotal lipid component in the serum of patients with severe spermatogenesis disorders. The sperm quality was examined, and histopathological analysis and biotin tracer tests were performed to assess spermatogenesis function and the integrity of the blood-testis barrier (BTB) in vivo. Cell-based in vitro experiments were carried out to investigate the effects of OCA administration on Sertoli cell dysfunction. This research aimed to elucidate the mechanism by which OCA may influence the function of Sertoli cells.

**Results:**

A pronounced reduction in OCA content was observed in the serum of patients with severe spermatogenesis disorders, indicating that OCA deficiency is related to spermatogenic disorders. The protective effect of OCA on reproduction was tested in a mouse model of spermatogenic disorder induced by busulfan at a dose 30 mg/kg body weight (BW). The mice in the study were separated into distinct groups and administered varying amounts of OCA, specifically at doses of 32, 64, 128, and 256 mg/kg BW. After evaluating sperm parameters, the most effective dose was determined to be 32 mg/kg BW. In vivo experiments showed that treatment with OCA significantly improved sperm quality, testicular histopathology and BTB integrity, which were damaged by busulfan. Moreover, OCA intervention reduced busulfan-induced oxidative stress and autophagy in mouse testes. In vitro, OCA pretreatment (100 µM) significantly ameliorated Sertoli cell dysfunction by alleviating busulfan (800 µM)-induced oxidative stress and autophagy. Moreover, rapamycin (5 µM)-induced autophagy led to Sertoli cell barrier dysfunction, while OCA administration exerted a protective effect by alleviating autophagy.

**Conclusions:**

This study demonstrated that OCA administration suppressed oxidative stress and autophagy to alleviate busulfan-induced BTB damage. These findings provide a deeper understanding of the toxicology of busulfan and a promising avenue for the development of novel OCA-based therapies for male infertility.

**Supplementary Information:**

The online version contains supplementary material available at 10.1186/s12944-024-02157-2.

## Introduction

Infertility has emerged as a significant global health concern, and male factors contribute to at least 50% of cases of infertility [1, 2]. Spermatogenesis disorders represent one of the most severe and intricate manifestations of male infertility. In recent decades, significant progress has been made by scientists toward comprehending the underlying causes and mechanisms associated with spermatogenesis disorders. Various adverse factors, including genetic mutations, environmental influences, and exposure to chemotherapy drugs, can contribute to spermatogenesis disorders [2–4]. Busulfan, a commonly used alkylating agent with nonspecific effects, is frequently given as a preparatory regimen before allogeneic hematopoietic stem cell transplantation. Studies have shown that it can lead to disruptions in male sperm production [5, 6]. Animal models induced by busulfan for studying spermatogenesis disorders are widely used to explore the mechanisms of new medications and therapies for male infertility [7].

Despite the availability of assisted reproductive technology (ART) as a viable approach to address specific fertility challenges, the universal applicability of these technologies remains limited [8]. Furthermore, despite the encouraging outcomes observed following the administration of recently identified compounds such as liver growth factor (LGF), melatonin, glial cell line-derived neurotrophic factor (GDNF), and others, rigorous investigations will be paramount to ascertain their long-term efficacy and safety, particularly in human subjects [9–11]. Consequently, there is an imperative need to discover novel therapeutic targets and enhance the efficacy of existing interventions for male infertility caused by spermatogenesis disorders.

Fatty acids are a category of organic compounds characterized by extended hydrocarbon chains. These biomolecules hold paramount significance within the body due to their roles in orchestrating multifaceted pathways, including contributing to the structural integrity of cellular membranes, modulating metabolic pathways, and actively participating in intricate physiological processes [12]. Recent investigations have predominantly focused on elucidating the beneficial effects of unsaturated fatty acids on the male reproductive system. Consuming omega-3 polyunsaturated fatty acids is thought to be important for preserving hormonal equilibrium, promoting the typical development of reproductive cells, and sustaining the structural integrity and motility of sperm [13–15]. Nevertheless, unsaturated fatty acids are inherently more prone to oxidation and heat-induced breakdown than saturated fats, which can make them more challenging to acquire [16]. Therefore, a more comprehensive exploration is warranted to identify a fatty acid that is easily accessible and beneficial to male reproductive function.

OCA is an eight-carbon medium-chain saturated fatty acid commonly found in coconut oil and dairy products [17, 18]. Several studies in humans have demonstrated the potential health benefits of OCA, including its ability to improve lipid metabolism and suppress inflammatory responses [19]. Additionally, OCA treatment has been shown to alleviate H_2_O_2_-induced oxidative stress, which can improve the development of liver disease [20]. Furthermore, OCA supplementation has been found to prevent lipopolysaccharide (LPS)-induced acute liver injury by upregulating autophagy [21]. Although OCA supplementation is potentially beneficial to human health, whether OCA plays a role in spermatogenesis and male reproductive health remains unclear.

Hypothesized from these inquiries, it can be argued that OCA has the potential to reduce busulfan-induced damage in spermatogenesis, leading to improvements in male fertility. To verify this hypothesis, the potential therapeutic effects of OCA supplementation were first investigated using a busulfan-induced mouse model of spermatogenesis disorder, and then, the sperm quality and BTB integrity were determined. These findings indicate that supplementation with OCA might ameliorate spermatogenesis disorders and BTB damage, highlighting its potential as a treatment for male infertility. Furthermore, the therapeutic effect of OCA can be attributed to its ability to alleviate busulfan-induced oxidative stress and autophagy. The findings presented in this study provide valuable insights into how busulfan contributes to reproductive toxicity and the potential for developing more effective treatments for male infertility.

## Materials and methods

### Analysis of human serum fatty acids and sperm quality

During the period from September 2017 to January 2020, serum specimens were collected from thirty subjects who experienced spermatogenesis abnormalities. This group consisted of 27 patients diagnosed with nonobstructive azoospermia (NOA) and 3 with extreme oligospermia (EO). Additionally, 30 individuals without any known fertility issues who were in the process of receiving standard semen assessments at the medical laboratory served as the control group. The participants in this study were between the ages of 20 and 43 (Supplementary Material 1, Table S1-S2), and blood samples were collected between 8:00 and 11:00 am. Subsequently, the samples were centrifuged at 1800×g for 10 min at room temperature to separate the serum. In previous research [22], the serum levels of free fatty acids were evaluated. Shanghai Applied Protein Technology Co. Ltd. utilized GC/MS to measure 39 medium- and long-chain free fatty acids (C6–C24). The sperm quality was assessed with a WLJY-9000 computer-assisted system (WLJY-9000, WeiLi, Beijing, China). The collection of clinical samples was conducted with informed consent and was authorized by the Research Ethics Committee of Jinling Hospital.

### Animals and experimental design

**Fig. 1:**
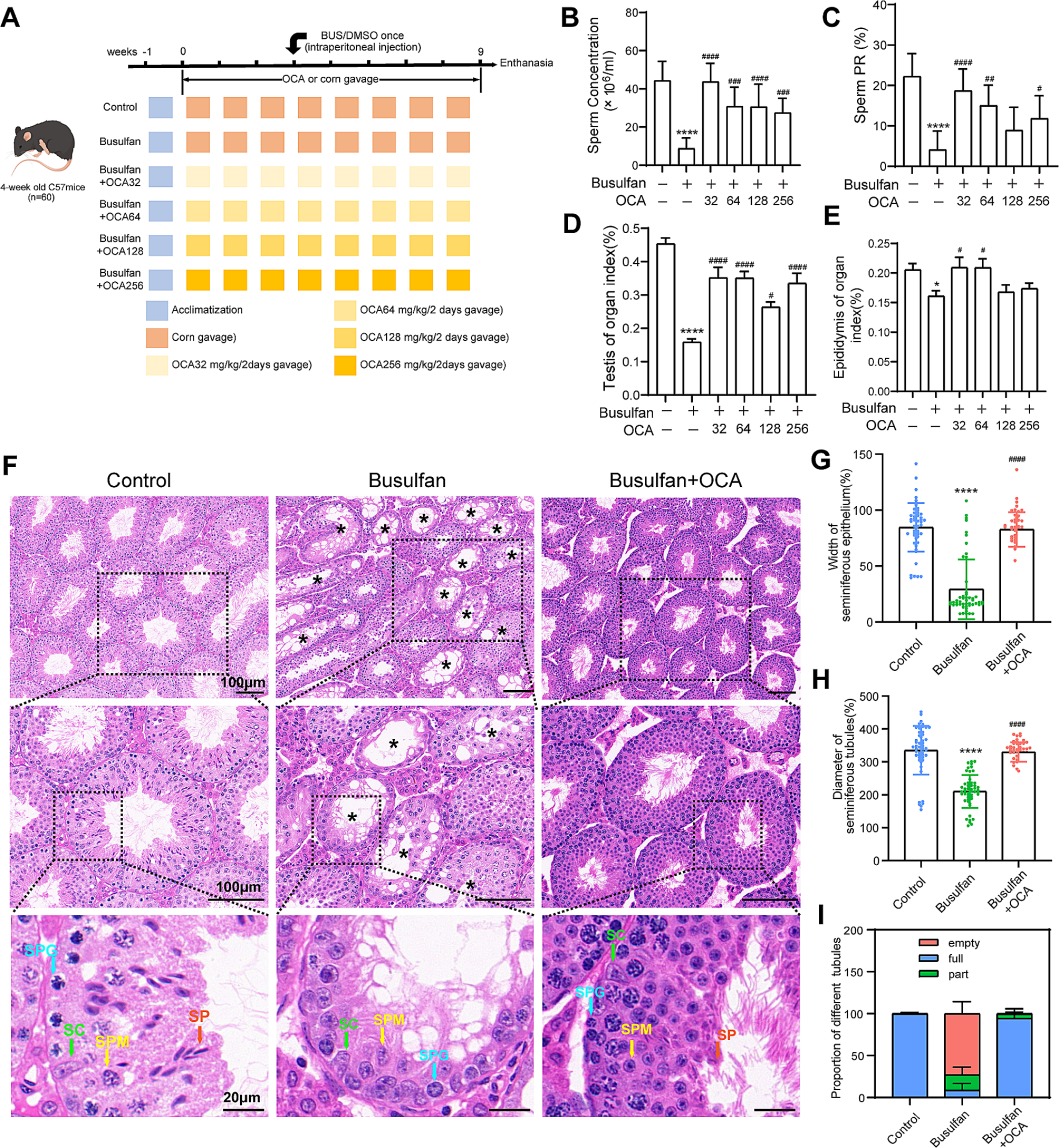
Experimental design and the effects of OCA and busulfan on spermatogenesis. (**A**) Experimental design. All animals were randomly divided into a control group, a busulfan group and four treatment groups with OCA (32, 64, 128, and 256 mg/kg BW every two days). (**B**-**E**) Sperm concentration (**B**), sperm PR (**C**), organ coefficient of the testis (**D**) and epididymis (**E**) of the mice at 9 weeks after OCA treatment (*n* = 7 ∼ 10). (**F**) Representative photographs of testicular morphology with HE staining. Scale bars: 100 μm and 20 μm. The black asterisk (*) indicates damaged spermatogenic tubules. SPG, spermatogonia; SPM, spermatocyte; SP, spermatozoa; SC, Sertoli cell. (**G**-**H**) The width of the seminiferous epithelium (**G**) and diameter of the seminiferous tubules (**H**) were calculated randomly from 50 cross-sections of round or nearly round seminiferous tubules (long axis: short axis < 1.2:1). (**I**) Proportion of different seminiferous tubules calculated from 10 random fields for each group. The data are presented as the mean ± SD. Statistical analyses were carried out using one-way ANOVA followed by Tukey’s post hoc test. ****P < 0.0001 vs. the control group, ^####^*P* < 0.0001 vs. the busulfan group

This study utilized 60 4-week-old male C57BL/6 mice obtained from Beijing Vital River Laboratory Animal Technology Co., Ltd. (Beijing, China) for the purpose of conducting in vivo experiments. Sigma-Aldrich (Missouri, USA) provided the OCA (C2875) and busulfan (55-98-1). Figure 1A depicts the experimental layout. Following a seven-day acclimatization period, the mice were distributed randomly into six categories: control, busulfan alone, and four OCA therapy sets at doses of 32, 64, 128, and 256 mg/kg BW, with each group containing 10 mice. The control group received Coil oil orally for 3 weeks and then received a single dimethylsulfoxide (DMSO) injection intraperitoneally. The busulfan alone group was given Coil oil orally for 3 weeks and a busulfan injection of 30 mg/kg BW intraperitoneally. The OCA + busulfan group received OCA in Coil oil orally, followed by a busulfan injection of 30 mg/kg BW intraperitoneally. OCA treatment continued for 5 weeks, after which the mice were euthanized for testis sample collection and subsequent analysis.

The initial busulfan concentration was 30 mg/mL in DMSO, which was thoroughly mixed with an equivalent volume of PBS in an ultrasonic device at 37 °C to reduce its toxicity. The mice were first euthanized using ether and then sacrificed through cervical dislocation to collect testis and epididymis samples. Postanesthesia cervical dislocation was considered a humane euthanasia method, as outlined in the Guide for the Care and Use of Laboratory Animals (Eighth Edition) by the Institutional Animal Care & Use Committee. The experimental protocols were conducted in adherence to the standards established by the National Laboratory Animal Care and Use Research Committee.

### TM4 cell treatment

To determine the best treatment concentrations of OCA and busulfan, busulfan was dissolved in DMSO, while OCA was dissolved in alcohol, and both were diluted in culture medium. Equal amounts of DMSO and alcohol were added to the control, busulfan, and OCA groups at a final concentration of less than 0.1% based on a previous study [23]. TM4 cells were either pretreated with OCA (100 µM) for 2 h or exposed to busulfan (800 µM) for 24 h. The optimal OCA concentration was established through the CCK-8 assay. Furthermore, in the rapamycin intervention experiments, TM4 cells were preexposed to OCA for 2 h before being subjected to rapamycin (5 µM) (S1842, Beyotime Biotechnology, Shanghai, China), as confirmed by the CCK-8 assay.

### Sperm quality analysis

The method for evaluating sperm quality was previously described [22]. The epididymides were recently transferred into human tubal fluid (HTF) medium and sliced into small fragments with ophthalmic scissors. Following incubation at 37 °C for 5 min, a hemocytometer (Qiujing, Shanghai, China) was used to observe 10 µL of the sperm suspension with the assistance of a light microscope (Olympus, Tokyo, Japan). The numbers of forward-moving sperm, nonforward-moving sperm, and immobile sperm were counted separately.

### Histology analysis of the testis and epididymis

The testis and epididymis samples were fixed by immersion in Bouin’s solution for 24 h, followed by dehydration using ethanol of varying concentrations. Subsequently, they were embedded in paraffin for sectioning. Five-micron-thick tissue sections were then stained with hematoxylin and eosin (HE) before being imaged and digitized under an optical microscope.

### BTB integrity analysis

The evaluation of BTB integrity was carried out with a biotin tracer, as detailed in previous studies [24, 25]. After receiving 5 weeks of busulfan treatment, three mice were chosen at random from each experimental group for additional analysis. Then, the mice were given 1% sodium pentobarbital injection at a dosage of 5 mL/kg BW via intraperitoneal administration for anesthetization. Incisions were created in the lower abdomen of the mice to expose their testes. Subsequently, 20 µL of freshly prepared EZ-Link Sulfo-NHS-LC-Biotin (21,335, Thermo Fisher Scientific, Massachusetts, USA) in PBS supplemented with 1 mM CaCl2 was injected into the stroma of the upper, middle, and lower testicular regions. The testes were extracted after 30 min of diffusion and promptly frozen in liquid nitrogen for cryosectioning. The slices, 10 μm in thickness, were immersed in 4% paraformaldehyde for 10 min and then treated with Alexa Fluor 488-labeled streptavidin (S32354, Thermo Fisher Scientific, Massachusetts, USA) for 1 h at room temperature. The specimens were then stained with DAPI and examined using a fluorescence microscope to visualize the seminiferous tubules. BTB damage was assessed using the following equation: A total of 50–60 round or oval-shaped cross-sections of the seminiferous tubules from each group were randomly examined. For oval-shaped tubules, *D*_*biotin*_ was computed as the average of the long and short axes of the tubule.

$$The\ extent\ of\ BTB\ damage=\frac{{D}_{biotin}}{{D}_{radius}}\times 100\%$$

*(D*_*biotin*_ *: the diffusion distance of biotin; D*_*radius*_ *: the radius of the tube)*

### Western blot

Testicular samples and cells were subjected to different treatments and lysed in RIPA buffer. The proteins were extracted by sonication on ice, followed by centrifugation at high speed and low temperature. Following the quantification of protein concentrations, proteins (20 µg) were resolved via SDS‒PAGE and subsequently transferred to PVDF membranes (Millipore, Massachusetts, USA). Following the blocking process with BSA, both primary and secondary antibodies were added to the membranes. The following primary antibodies were used in the present study: ZO-1 rabbit pAb (21773-1-AP, ProteinTech, Wuhan, China), Claudin11 rabbit pAb (AF5364, Affinity, Ohio, USA), Claudin5 rabbit pAb (AF5216, Affinity, Ohio, USA), Occludin rabbit pAb (13409-1-AP, ProteinTech, Wuhan, China), HO1/HMOX1 rabbit pAb (10701-1-AP, ProteinTech, Wuhan, China), NQO1 rabbit pAb (11451-1-AP, ProteinTech, Wuhan, China), SQSTM1/P62 rabbit mAb (ab109012, Abcam, Shanghai, China), and LC3B rabbit mAb (ab192890, Abcam, Shanghai, China). The visualization of the target protein bands was carried out using a chemiluminescent imaging system. The protein bands were subsequently quantified using ImageJ.

### Analysis using real-time PCR

Gene expression levels related to oxidative stress were measured through RT‒PCR analysis in this study. After total RNA was isolated from the testes using a Total RNA Purification Kit (082001, BEI-BEI Biotech, Zhengzhou, China), cDNA synthesis was conducted utilizing HiScript III RT SuperMix for qPCR (R323, Vazyme Biotechnology, Nanjing, China) following the evaluation of RNA concentration and integrity. The quantitative analysis was carried out on a Roche LightCycler 96 Real-time PCR machine (Roche Diagnostics, Basel, Switzerland) using qPCR SYBR Green Master Mix (Q121, Vazyme Biotechnology, Nanjing, China). The reference gene β-actin served for uniform labeling, and gene expression values were calculated using the 2-ΔΔCq formula [26]. The sequences of primers used in the present study are listed in Table S3 (Supplementary Material 1).

### Measurement of SOD and MDA

In brief, the tissue samples were homogenized and then centrifuged to extract protein. Following the prescribed protocol, the activity of SOD and the concentration of MDA were measured using specific assay kits.

### Intracellular ROS quantification

The levels of ROS within the cells were quantified utilizing a DCFH-DA fluorescent probe (S0033 M, Beyotime Biotechnology, Shanghai, China) followed the manufacturer’s guidelines. Following treatment, 10 µM DCFH-DA solution in medium devoid of FBS was added to each corresponding well. Afterward, the cells were incubated in a lightless setting at 37 °C for 20 min prior to being rinsed twice with medium devoid of FBS. The brightness of the fluorescence was captured with a fluorescence microscope.

### Transepithelial electrical resistance (TER) evaluation

To evaluate cell barrier performance in a controlled environment, TER was measured daily in three specific regions of the samples using a Milli-cell electrical resistance system (Millipore, Massachusetts, USA). Sertoli cells were initially inoculated in MilliCell Hanging Cell Culture Inserts (PET 0.4 μm, Millipore, Massachusetts, USA) at a concentration of 0.5 × 10^6^ cells/cm^2^ and allowed to develop for three days to establish cellular barriers. After treatment, a Millicell electrical resistance system (Millipore, Massachusetts, USA) was used to measure the TER. The TER was calculated by the following formula: TER (Ω·cm^2^) = (resistance from treatment (Ω) - initial resistance (Ω)) × surface area of membrane (cm^2^).

### Statistical analysis

The data illustrated in this investigation were sourced from a minimum of three distinct in vivo specimens and three separate in vitro trials. The outcomes were visualized utilizing GraphPad Prism 7 (GraphPad Software, California, USA) and are reported as the means ± standard deviations (SDs). Statistical significance across various groups was scrutinized utilizing SPSS 19.0 software (SPSS, Illinois, USA) through independent t tests and one-way analyses of variance (ANOVAs) followed by post hoc assessments of least significant divergence (matching variances) or Games-Howell (mismatching variances). A significance level of *P* < 0.05 was used to determine statistical significance.

## Results

### Serum OCA levels are significantly lower in azoospermic patients

**Fig. 2:**
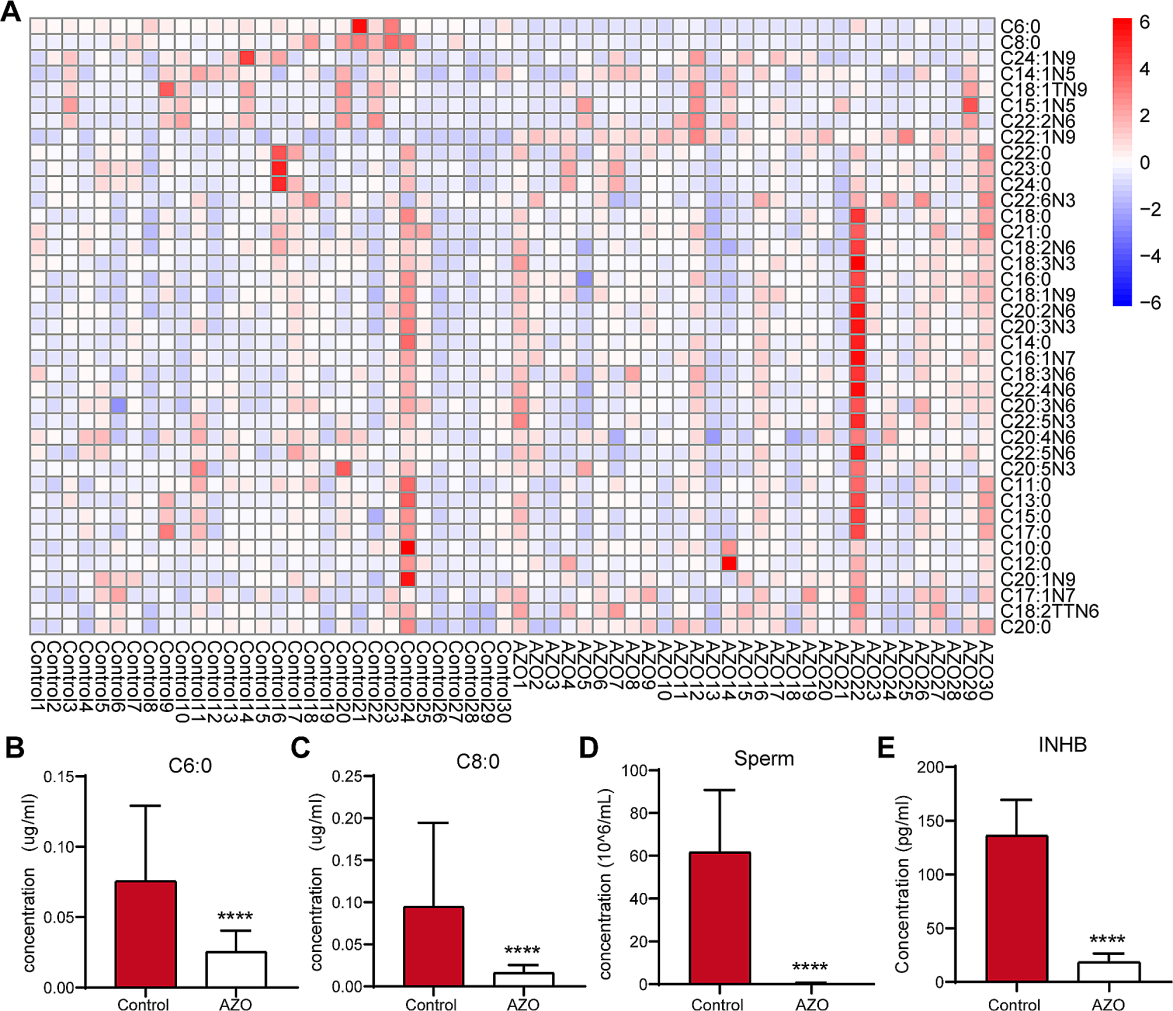
Analysis of fatty acid and INHB levels in the serum and sperm. (**A**) Heatmap of the serum fatty acid composition. Control, healthy individuals (*n* = 30); AZO, patients with azoospermia (*n* = 30); AZO26, 28 and 30 represent EO, while the others represent NOA. (**B**-**C**) Fatty acid levels of C6 (**B**) and C8 (**C**) in serum. (**D**) Sperm concentration. (**E**) Serum INHB levels. INHB, inhibin B. The data are presented as the mean ± SD. Statistical analyses were carried out using two-tailed Student’s t tests (****P < 0.0001). EO, extreme oligospermia; NOA, nonobstructive azoospermia

To identify the potential fatty acids that are potentially beneficial to male reproductive function, a comprehensive clinical analysis was performed. This analysis involved a comparative examination of the fatty acid compositions (C6-C24) of serum samples from two cohorts—30 healthy individuals and 30 patients—diagnosed with azoospermia (AZO), including NOA and EO. Through the application of GC/MS for quantifying the serum concentrations of 39 medium- and long-chain fatty acids, notable modifications were identified in the fatty acid composition within the serum of individuals compared with controls (Fig. 2A), revealing a marked reduction in the levels of C6 and C8 fatty acids with statistical significance (*P* < 0.0001) (Fig. 2B-C). Furthermore, a greater decrease in the levels of OCA (C8) was noted in this study (Fig. 2C), indicating that supplementation with OCA could improve spermatogenesis. At the same time, there was a notable reduction (*P* < 0.0001) in the production of inhibin B (INHB) (Fig. 2E), a reproductive hormone released by Sertoli cells, in the plasma of these individuals, whose sperm count exhibited a corresponding decline (*P* < 0.0001) (Fig. 2D). These findings from the medical evaluation suggest a potential link between OCA deficiency and impaired Sertoli cell function.

### OCA improves spermatogenesis disorders in busulfan-treated mice

To explore whether OCA supplementation can ameliorate spermatogenesis disorders and Sertoli cell dysfunction, a mouse model of impaired spermatogenesis was induced using a single intraperitoneal dose of busulfan. OCA was subsequently given to these mice orally following the procedure depicted in Fig. 1A. Previous investigations have demonstrated that the highest dose of OCA (128 mg/kg BW) did not cause dose-limiting toxicity, and the most common adverse event observed was mild abdominal discomfort, indicating that administration of OCA via gavage is safe and reliable [27]. Therefore, four concentrations of OCA (32, 64, 128, and 256 mg/kg BW) were used to investigate the potential therapeutic effects of OCA via gavage (Fig. 1A).

Compared to the control group, the OCA-receiving group exhibited a notable reduction in the sperm concentration (*P* < 0.0001), forward movement of sperm (*P* < 0.0001), weight of the testis (*P* < 0.0001) and epididymis (*P* < 0.01), and coefficients of organs, including the testis (*P* < 0.0001) and epididymis (*P* < 0.05) (Fig. 1B-E, Fig. S1A-B), indicating the induction of spermatogenesis disorders by busulfan. However, mice administered each of the four doses of OCA (32, 64, 128, or 256 mg/kg BW) displayed significantly elevated sperm concentrations, sperm progressive motility, testis and epididymis weights, and organ coefficients of the testis and epididymis relative to mice solely receiving busulfan (Fig. 1B-E, Fig. S1A-B), suggesting the successful amelioration of busulfan-induced spermatogenesis disorder through OCA administration. Furthermore, histological analysis of epididymal tubules using hematoxylin and eosin (HE) staining revealed a significant decrease in sperm density in the epididymal cauda of the mice in the busulfan group, which was also restored by OCA supplementation (Fig. S1C). Based on these results, it can be inferred that a clear therapeutic effect existed in the groups treated with OCA, and a better effect was observed at the lower concentration (32 mg/kg BW). Therefore, most of the following experiments were performed using this dose.

To determine the protective effects of OCA supplementation on testicular health, the histopathology of the testes was evaluated using HE staining. Figure 1F illustrates that the testicular morphology of the busulfan group exhibited noticeable changes in the seminiferous tubules, including atrophy, vacuolation, and loss of germ cells. Conversely, the group supplemented with OCA showed well-organized seminiferous tubules and the reemergence of germ cells. Additionally, the dimensions of the seminiferous tubules and the thickness of the seminiferous epithelium were both measured. A notable decline (*P* < 0.001) in these variables was noted in the busulfan-treated group, whereas a rise (*P* < 0.001) was observed in the group treated with OCA (Fig. 1G-H). Furthermore, there was a notable increase in abnormal tubules (including empty and partial types) after busulfan administration, but this proportion was notably reduced in the OCA-treated group (Fig. 1I). These findings suggest that OCA supplementation can expedite the regeneration of spermatogenesis impaired by busulfan treatment in mice.

### OCA supplementation restores busulfan-disrupted BTB integrity in mouse testes

**Fig. 3:**
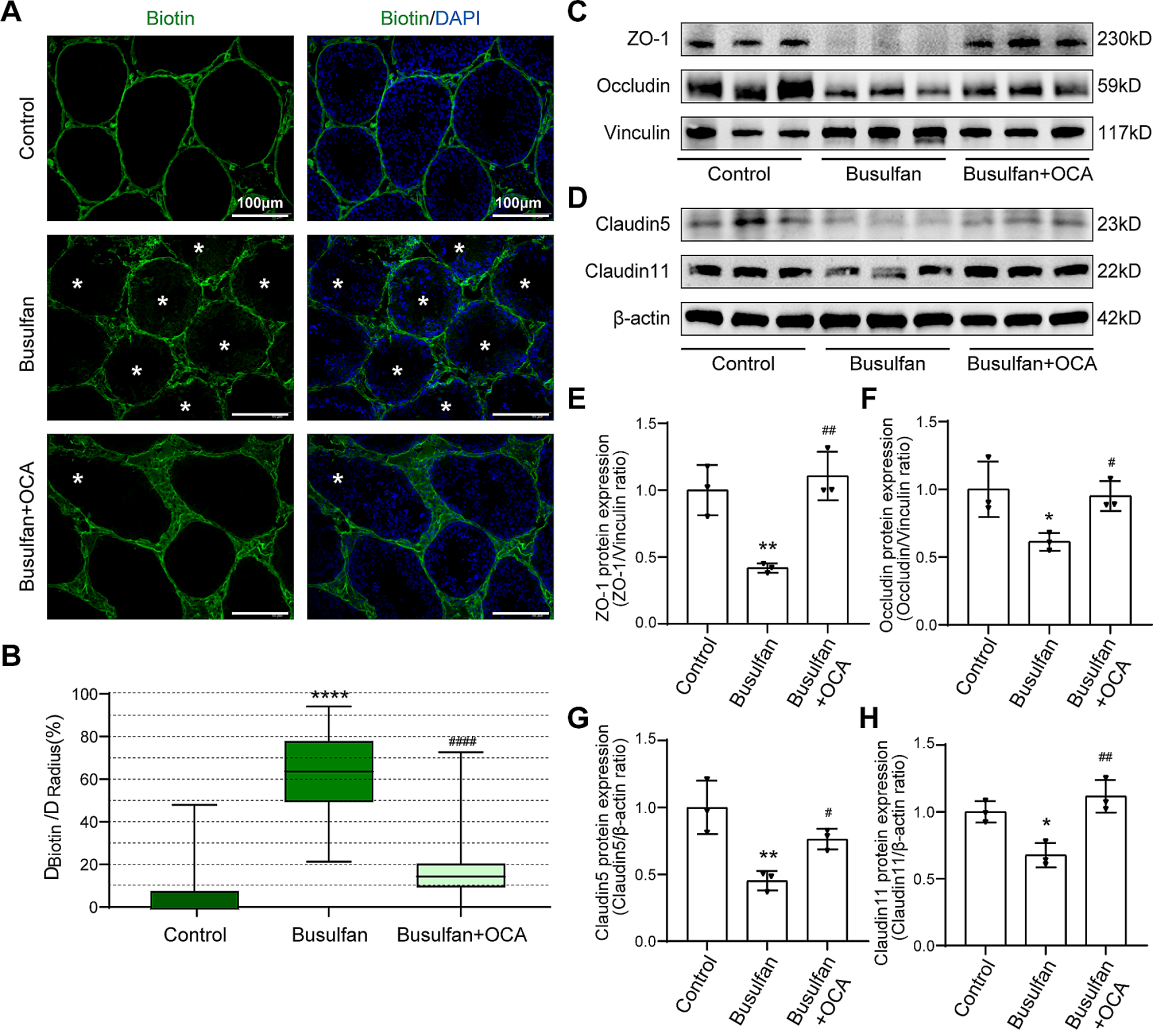
Effects of OCA and busulfan on the BTB in mice. (**A**) Representative fluorescence images of BTB integrity detected by the biotin tracer assay. The white asterisk (*) indicates the permeation of biotin into the seminiferous lumen. Scale bars: 100 μm. (**B**) Box plot illustrating the extent of BTB damage, which was calculated randomly from 50–60 seminiferous tubules for each group. (**C**-**D**) Representative images of tight junction proteins were examined by western blotting in mouse testes (*n* = 3). (**E**-**H**) The relative protein expression of ZO-1 (**E**), Occludin (**F**), Claudin5 (**G**) and Claudin11 (**H**) was quantified by ImageJ and normalized to vinculin or β-actin levels (*n* = 3). The data are presented as the mean ± SD. Statistical analyses were carried out using one-way ANOVA followed by Tukey’s post hoc test. *P < 0.05, **P < 0.01, ****P < 0.0001 vs. the control group; ^#^*P* < 0.05, ^##^*P* < 0.01, ^####^*P* < 0.0001 vs. the busulfan group. ZO-1, zona occluden-1

To validate whether OCA supplementation could alleviate the effects of busulfan toxicity on spermatogenesis by restoring Sertoli cell function, a semiquantitative in vivo assay in which biotin was used as an indicator was performed. As demonstrated in Fig. 3A-B, a fully functional BTB obstructed the entry of biotin into the seminiferous lumen. This led to the detection of streptavidin-488 fluorescence solely within the testicular interstitium and basal membranes of the control group mice.

In contrast, injection of busulfan resulted in a greater permeation distance of biotin by approximately 60% (*P* < 0.0001), while supplementation with OCA reduced the leakage of biotin by approximately 17% (*P* < 0.001), indicating that OCA treatment restored BTB integrity. Busulfan administration also significantly reduced the levels of the BTB-related connexins ZO-1, Occludin, Claudin5 and Claudin11 (*P* < 0.01, *P* < 0.05, *P* < 0.01, *P* < 0.05, respectively) (Fig. 3C-H). Conversely, treatment with OCA led to a noticeable increase in the expression of these proteins (*P* < 0.01, *P* < 0.05, *P* < 0.05, *P* < 0.01) (Fig. 3C-H). These results indicate that OCA may hold promise for ameliorating the Sertoli cell dysfunction observed in mice treated with busulfan.

### OCA alleviates oxidative stress and autophagy induced by busulfan in mouse testes

**Fig. 4:**
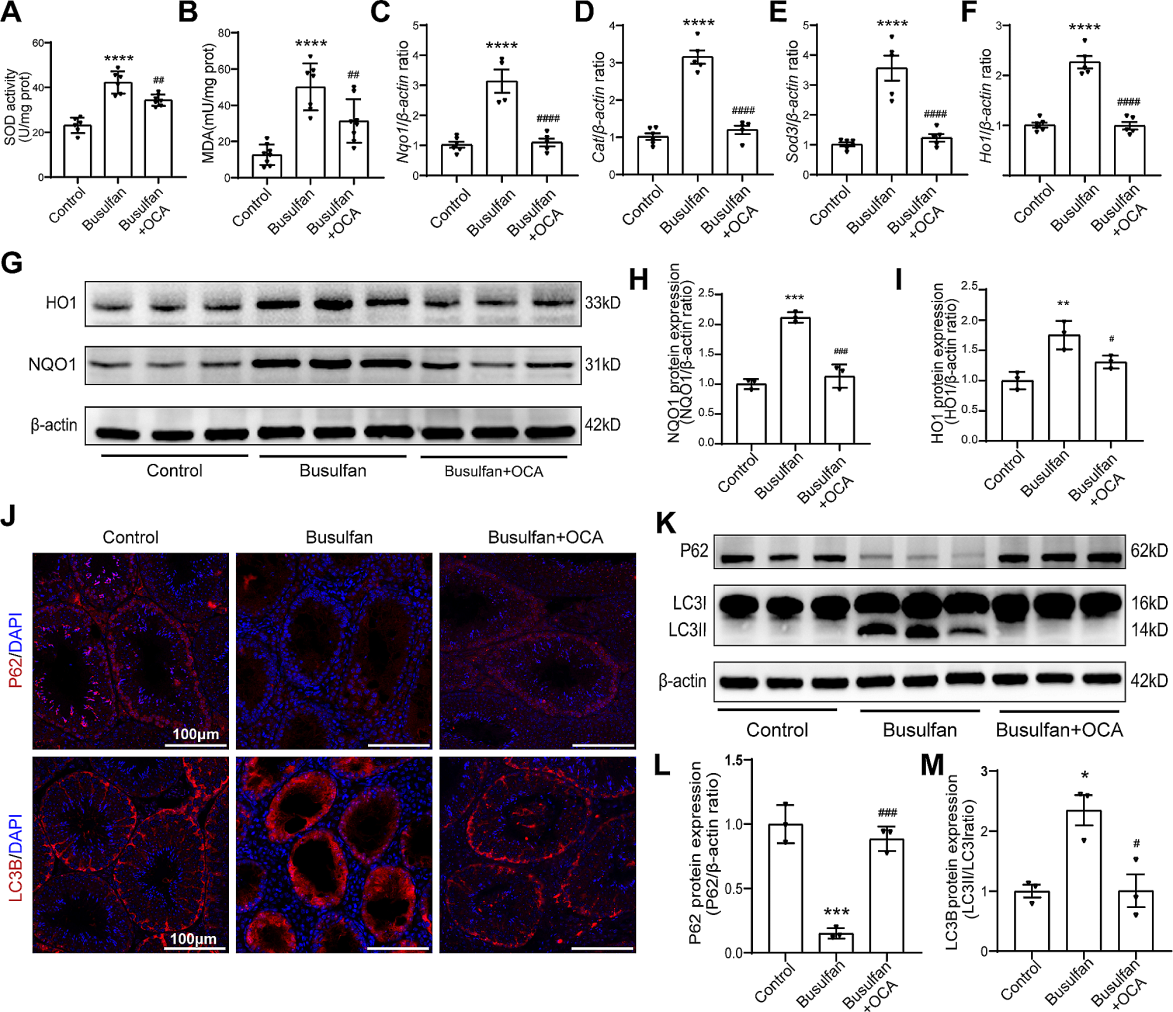
Changes in oxidative stress levels in the testis after busulfan injection with or without OCA supplementation. (**A**-**B**) SOD activity and MDA levels in the testes of mice (*n* = 6 ∼ 8). (**C**-**F**) The mRNA levels of NQO1 (**C**), CAT (**D**), SOD3 (**E**) and HO1 (**F**) were examined by qPCR and normalized to β-actin levels. (*n* = 4 ∼ 6). (**G**) Representative images of HO1 and NQO1 protein expression in testes were examined by western blotting. (**H**-**I**) The protein expression levels of HO1 (**H**) and NQO1 (**I**) were quantified by ImageJ and normalized to β-actin levels (*n* = 3). (**J**) Representative immunofluorescence images of P62 (red) and LC3B (red) in the testis. Scale bar: 100 μm. (**K**) Representative images of LC3B and P62 protein expression in testes were examined by western blotting (*n* = 3). (**L**-**M**) The protein expression levels of P62 (**L**) and LC3B (**M**) were quantified by ImageJ and normalized to β-actin levels (*n* = 3). The data are presented as the mean ± SD. Statistical analyses were carried out using one-way ANOVA followed by Tukey’s post hoc test. *P < 0.05, ***P < 0.001 vs. the control group; ^#^*P* < 0.05, ^###^*P* < 0.001 vs. the busulfan group. SOD: superoxide dismutase; CAT, catalase; NQO1: NAD(P)H, quinone oxidoreductase 1; HO1, heme oxygenase 1; P62, SQSTM1, sequestosome 1; LC3, light chain 3

Previous studies have demonstrated that OCA can increase antioxidant capacity and regulate autophagy to benefit the functions of biological systems [20, 21, 28]. To explore whether OCA could alleviate oxidative stress levels to restore Sertoli cell dysfunction in vivo, testicular SOD activity and MDA levels, which were significantly increased (*P* < 0.0001) in the busulfan-treated group and decreased (*P* < 0.01) in mice after OCA treatment, were assessed (Fig. 4A-B). Additional studies were carried out to assess oxidative stress-related gene expression in the testes. The mRNA levels of *Nqo1*, *Ho1*, *Cat*, and *Sod3* were significantly increased (*P* < 0.001) by busulfan treatment but decreased (*P* < 0.001) after OCA supplementation (Fig. 4C-F). Additionally, compared with busulfan treatment, OCA treatment significantly decreased the protein levels of NQO1 and HO1 (*P* < 0.05, *P* < 0.001) (Fig. 4G-I). In addition, the levels of the autophagy-associated proteins LC3B and P62, which are involved in autophagosome formation and breakdown, respectively, were assessed. Immunofluorescence staining demonstrated the presence of both LC3 and P62 in the Sertoli and spermatogenic cell layers in the control group (Fig. 4G). After exposure to busulfan, there was a marked increase in the LC3-II/LC3-I ratio (*P* < 0.05) and a significant decrease in P62 levels (*P* < 0.001) (Fig. 4K-M), indicating that busulfan can trigger autophagy and enhance autophagic breakdown in the testes. Conversely, supplementation with OCA resulted in contrasting trends in the LC3-II/LC3-I ratio and P62 expression levels compared to those in the busulfan group (Fig. 4K-M). These findings imply that OCA could mitigate the oxidative stress and autophagy caused by busulfan in the testes, thereby promoting the restoration of spermatogenesis and Sertoli cell function.

### OCA supplementation alleviates busulfan-induced oxidative stress and autophagy in TM4 sertoli cells

**Fig. 5:**
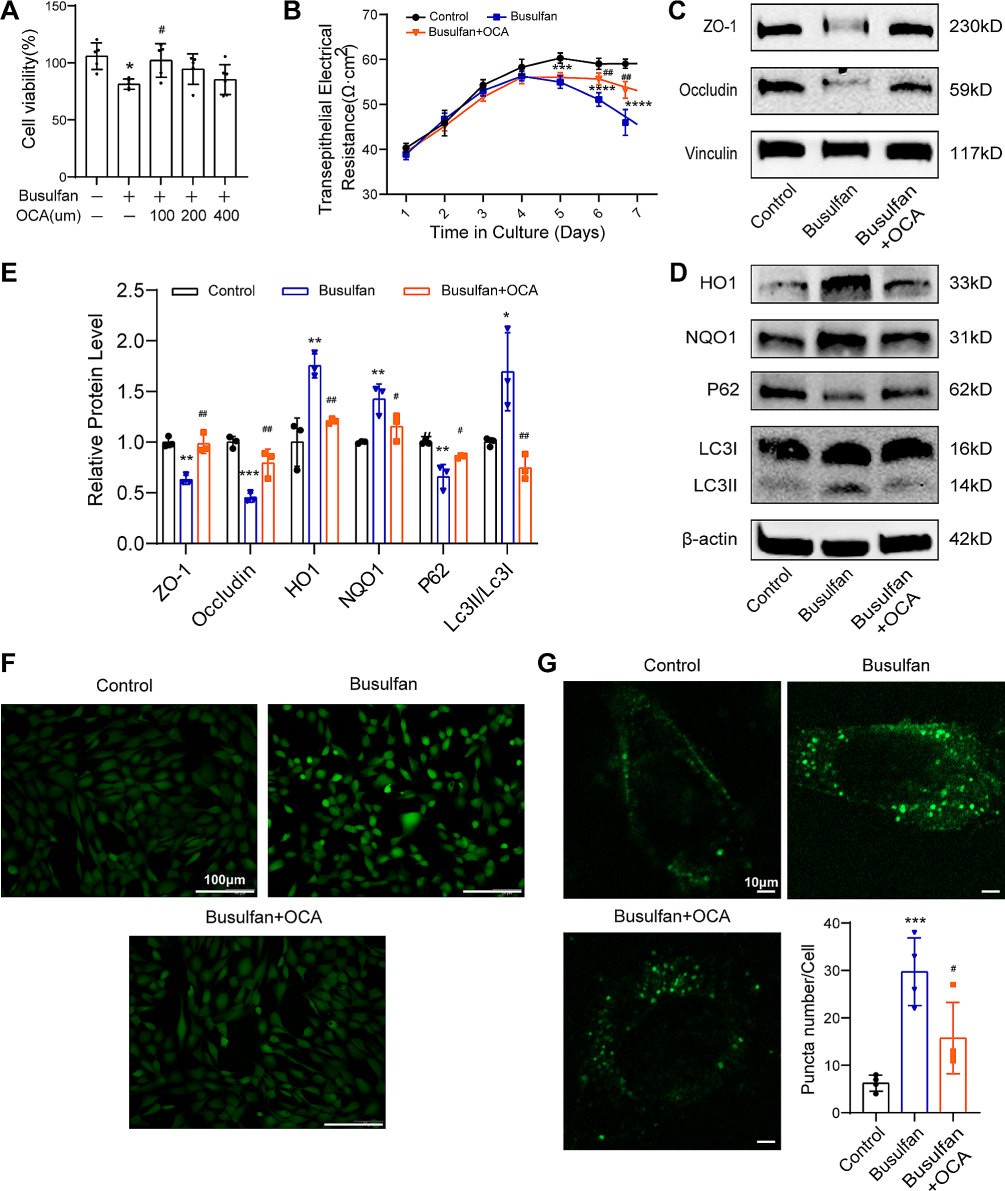
Effects of OCA and busulfan on oxidative stress in TM4 cells. (**A**) Analysis of the cell survival-rescuing effects of OCA after busulfan treatment by CCK-8 (*n* = 5). (**B**) TER detection of TM4 cell barriers (*n* = 4). (**C**-**D**) Representative images of ZO-1, Occludin, HO1, NQO1, LC3B and P62 proteins were examined by western blotting. (**E**) The relative protein expression levels were quantified by ImageJ and normalized to those of β-actin or vinculin (*n* = 3). (**F**) Representative images of ROS generation in the busulfan group and OCA pretreatment group. Scale bar: 100 μm. (**G**) Representative images of the formation of autophagosomes by GFP-LC3 immunofluorescence; scale bar = 10 μm. Quantification of the number of GFP-positive puncta in each TM4 cell line (*n* = 4). The data are presented as the means ± SDs. Statistical analyses were carried out using one-way ANOVA followed by Tukey’s post hoc test. *P < 0.05, **P < 0.01, ***P < 0.001, ****P < 0.0001 vs. the control group; ^#^*P* < 0.05, ^##^*P* < 0.01 vs. the busulfan group. ZO-1, zona occluden-1; NQO1: NAD(P)H, quinone oxidoreductase 1; HO1, heme oxygenase 1; P62, SQSTM1, sequestosome 1; LC3, light chain 3

This study employed the Sertoli cell line TM4 to investigate the efficacy of OCA supplementation in reversing dysfunction in Sertoli cells by shielding them from oxidative stress and hindering autophagy in vitro. Cytotoxicity was initiated by subjecting TM4 cells to 800 µM busulfan, followed by treatment with varying therapeutic concentrations of OCA (100, 200, or 400 µM). Cell viability was assessed through a CCK-8 assay, as illustrated in Fig. 5A. Interestingly, the administration of 100 µM OCA led to a noteworthy increase (*P* < 0.05) in cell viability. Therefore, subsequent therapeutic experiments were conducted in vitro using this dose. Transepithelial electrical resistance (TER) assays revealed that compared with busulfan treatment, OCA treatment notably increased (*P* < 0.01) electrical resistance (Fig. 5B), indicating that OCA treatment protected against cell barrier damage caused by busulfan. Additionally, OCA therapy effectively reduced the decrease in the expression of BTB-related proteins (ZO-1 and Occludin) triggered by busulfan (Fig. 5C and E). Moreover, the protein levels of HO1, NQO1, P62 and LC3B were analyzed in TM4 Sertoli cells (Fig. 5D-E). OCA intervention alleviated the reduction in P62 protein levels and the elevation in HO1 and NQO1 levels, as well as the increase in the LC3II/LC3I ratio, compared to those in the busulfan-exposed group (Fig. 5D-E). Additionally, compared to busulfan treatment alone, OCA treatment significantly decreased the intracellular ROS levels and the number of cytoplasmic GFP-positive puncta (Fig. 5F-G). This finding suggested that OCA treatment effectively mitigated the oxidative stress and autophagy triggered by busulfan in vitro, consistent with the outcomes of the in vivo trials. These results suggest that OCA can improve busulfan-induced Sertoli cell dysfunction through the suppression of oxidative stress and autophagy.

### OCA treatment alleviates TM4 cell damage by suppressing autophagy

**Fig. 6:**
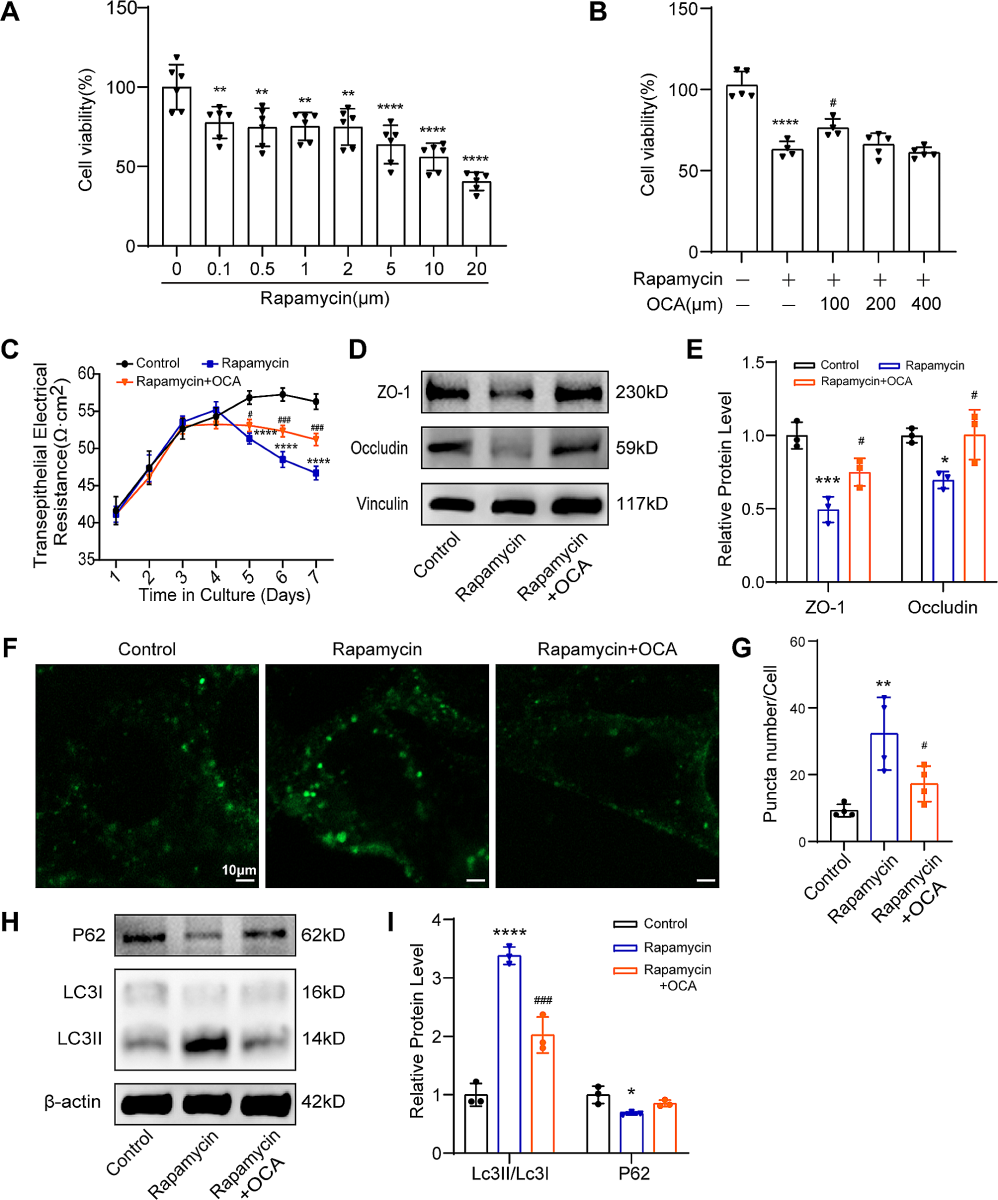
Effect of rapamycin treatment with or without OCA supplementation on autophagy in TM4 cells. (**A**) Analysis of cell viability after treatment with rapamycin (0, 0.1, 0.5, 1, 2, 5, 10, or 20 µM) for 24 h (*n* = 6). (**B**) Analysis of the cell survival-rescuing effects of OCA (100, 200, or 400 µM) after rapamycin treatment by CCK-8 (*n* = 5). (**C**) TER detection of TM4 cell barriers (*n* = 4). (**D**) Representative images of ZO-1 and Occludin proteins were examined by western blotting. (**E**) The relative protein expression levels were quantified by ImageJ and normalized to the vinculin levels (*n* = 3). (**F**) Representative images of the formation of autophagosomes by GFP-LC3 immunofluorescence; scale bar = 10 μm. (**G**) Quantification of the number of GFP-positive puncta in each TM4 cell line; *n* = 4. (**H**) Representative images of the LC3B and P62 proteins were examined by western blotting. (**I**) The relative protein expression levels were quantified by ImageJ and normalized to the β-actin levels (*n* = 3). For A and B, statistical analyses were carried out using one-way ANOVA followed by Dunnett’s multiple comparisons test. For C-F, statistical analyses were carried out using one-way ANOVA followed by Tukey’s post hoc test. The data are presented as the mean ± SD. *P < 0.05, **P < 0.01, ****P < 0.0001 vs. the control group; ^#^*P* < 0.05, ^###^*P* < 0.001 vs. the busulfan group. P62, SQSTM1, sequestosome 1; LC3, light chain 3; ZO-1, zona occluden-1

To validate the role of autophagy in Sertoli cell barrier integrity and the therapeutic effect of OCA, rapamycin, an autophagy activator, was used to induce autophagy in vitro. Based on the results of the CCK-8 assay (Fig. 6A-B), 5 µM rapamycin and 100 µM OCA were used in the in vitro intervention assay. In vitro experiments showed that rapamycin, similar to busulfan, had a significant negative effect on Sertoli cell barrier function (Fig. 6C-E), indicating that activating autophagy can disrupt this important barrier. Additionally, in the OCA-treated group, there was a notable increase in electrical resistance and elevated protein levels of ZO-1 and Occludin compared to those in cells treated only with rapamycin. These findings strongly indicate that OCA plays a pivotal role in restoring Sertoli cell barrier function. Furthermore, compared with rapamycin treatment, supplementation with OCA induced a significant decrease in GFP-LC3 fluorescence and the LC3II/LC3I ratio (Fig. 6F-G) and an increase in P62 expression (Fig. 6H-I). These findings indicate that OCA treatment can rescue Sertoli cell barrier damage by inhibiting autophagy.

**Fig. 7:**
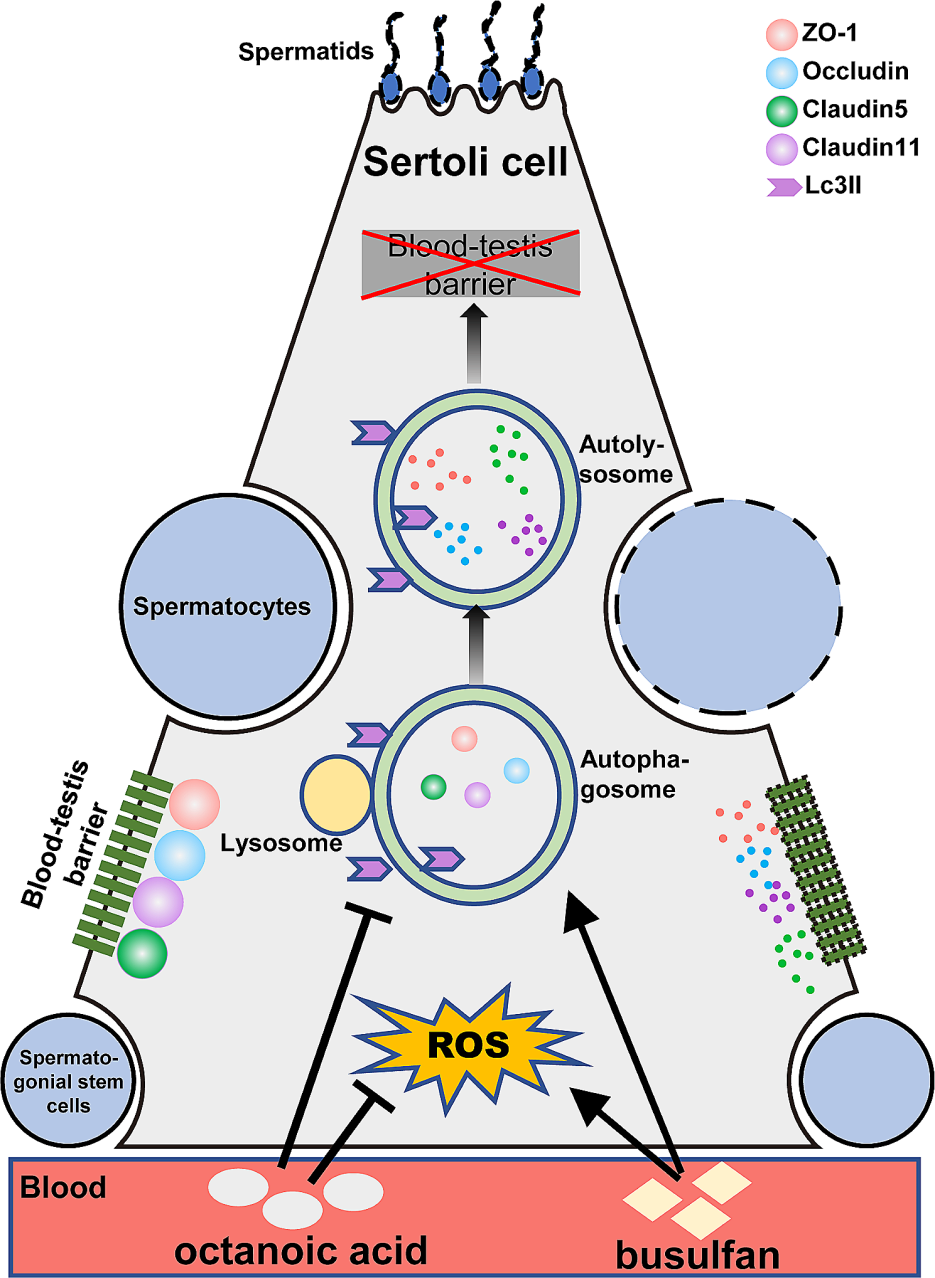
Mechanistic diagram of OCA and busulfan regulating Sertoli cell barrier function. Busulfan induces oxidative stress and autophagy in Sertoli cells, leading to downregulation of tight junction protein expression and ultimately disruption of the Sertoli cell barrier. Conversely, OCA alleviated oxidative stress and autophagy to ameliorate Sertoli cell dysfunction induced by busulfan. OCA, octanoic acid; ROS, reactive oxygen species; ZO-1, zona occluden-1; LC3II, light chain 3 II

## Discussion

In the course of this study, it was observed that individuals suffering from severe dyszoospermia exhibited a decrease in serum levels of OCA. This observation implies that supplementation with OCA could ameliorate spermatogenesis disorders (Fig. 2). In this study, OCA supplementation rescued spermatogenesis by reducing oxidative stress and autophagy levels in Sertoli cells.

Previous research has indicated that busulfan could inhibit spermatogenesis by harming germ cells directly or interfering with the BTB, resulting in male infertility [25, 29]. A recent investigation by Zhao et al. suggested that Sertoli cells are vital in the progression of dyszoospermia [30]. An essential role of Sertoli cells is to establish the BTB, creating a specific environment for germ cell growth that is shielded from potentially harmful compounds and immune reactions to sperm antigens [31]. In this study, OCA supplementation restored busulfan-induced damage to BTB integrity (Fig. 3). In addition, the serum INHB levels were decreased in patients with clinical spermatogenic dysfunction (Fig. 2E). Additional studies have demonstrated that busulfan has adverse effects on Sertoli cells [32]. Given the above findings, research on the impact of OCA has focused on Sertoli cells. Notably, this study revealed the beneficial effects of OCA on Sertoli cells and the BTB.

Oxidative stress is a significant factor in male infertility, and an imbalance of ROS is often cited as a main cause [33]. This imbalance has also been identified as a key contributor to busulfan-induced reproductive toxicity [34]. Accumulating in vitro and in vivo evidence has elucidated the multifaceted mechanisms by which OCA ameliorates various diseases, notably through the alleviation of oxidative stress [20, 35]. In this study, OCA treatment effectively improved busulfan-induced oxidative stress in the testes. Additionally, an in vitro study using TM4 cells confirmed the ability of OCA to mitigate busulfan-induced oxidative stress.

Autophagy, a lysosomal catabolic mechanism present in all eukaryotes, is essential for maintaining a balanced cellular environment by degrading proteins and organelles [36]. This process is intimately linked to male reproduction [37], and dysregulation of autophagy in Sertoli cells has been linked to male infertility and testicular dysfunction, including BTB destruction [38]. Modulating autophagy is being explored as a promising therapeutic approach for treating Sertoli cell dysfunction. Recent studies have shown that enteral nutrition rich in OCA can prevent acute liver injury induced by LPS through the upregulation of autophagy [21]. Therefore, autophagy may represent a potential mechanism by which OCA can improve Sertoli cell dysfunction. Furthermore, supplementation with OCA alleviated autophagy induced by busulfan or an autophagy agonist (Figs. 4, 5 and 6), indicating its potential protective effects against BTB damage. These findings highlight the therapeutic value of targeting autophagy for treating male infertility.

Contrary to the results presented in this study, Wei and colleagues observed that busulfan suppresses autophagy by halting the breakdown of autophagosomes, leading to the impairment of spermatogonial cells [39]. However, this finding does not contradict this study, as the effect of autophagy on male fertility is twofold [40]. Optimal autophagy can shield cells effectively from unfavorable environmental conditions, such as hypoxia, a lack of nutrients, oxidative stress, or the buildup of misfolded proteins. In times of stress, the upregulation of autophagy enables the cell to acclimate to changing surroundings and ensure its survival [41]. Nonetheless, excessive autophagy can result in the excessive consumption of proteins, harm to cell organelles, and impairment of cellular function, ultimately leading to defects in spermatogenesis and damage to the BTB [42–44]. This discovery implies that the status of autophagy may differ under different treatments. In conjunction with the current study, these findings underscore the importance of autophagy in busulfan toxicology, as well as the protective influence of OCA through the regulation of autophagy.

Notably, in this investigation, no dose‒response correlation was found with OCA, as mice receiving the lowest experimental dosage (32 mg/kg BW) showed marked enhancements. Remarkably, adding high doses of OCA has been found to have adverse impacts on the growth of blastocysts, rates of hatching, speed of development, and overall cell count [45]. Therefore, it can be speculated that the administration of OCA is a double-edged sword and that high doses of OCA may have adverse effects on spermatogenesis development in mice, indicating that the amount of OCA intake should be moderate. When OCA is used for the treatment of dyszoospermia, its dose should be precisely controlled to prevent its side effects.

Because of its reduced molecular size, OCA can be absorbed directly from the intestines, bypassing the lymphatic system and entering the liver through the portal vein directly [46]. Studies have demonstrated that the concentration of OCA in plasma increases after oral OCA administration [27]. According to a radiolabeled fatty acid tracer study, OCA can be rapidly transported into the central nervous system following oral administration [47]. These findings indicate that OCA can possibly reach the testes through the blood circulation and exert its effects on the spermatogenesis microenvironment. In vitro experiments confirmed the antioxidative and antiautophagic effects of OCA on Sertoli cells. This, in turn, leads to an improvement in Sertoli cell barrier function, consistent with the outcomes of in vivo gavage studies. In addition, OCA can also be metabolized into small molecule substances (e.g., ketone bodies) or synthesized into long-chain fatty acids to exert its biological functions [48, 49]. With respect to the functions of its metabolites, further investigations following a comprehensive analysis of the fatty acid composition of the local testicular milieu will improve the understanding of how OCA works in vivo.

### Strengths and limitations

The present study demonstrated that OCA could improve spermatogenesis by safeguarding Sertoli cells. This is the first study to investigate the therapeutic mechanism of OCA on male infertility. The amelioration of oxidative stress and autophagy was found to be the primary effects of OCA on Sertoli cells, which suggested that OCA could be utilized as a promising dietary intervention to enhance spermatogenesis in patients with male infertility.

However, in this research, the impacts of OCA were predominantly evaluated on Sertoli cells, while the effects of OCA on germ cells must not be disregarded and warrant future examination. If circumstances allow, single-cell omics may be utilized to examine the impacts of OCA on all cell types within the testis. Despite the results of this research supporting the idea that OCA could enhance spermatogenesis by mitigating oxidative stress and autophagy in Sertoli cells, the specific bioactive substances and metabolic components responsible for these therapeutic effects are still unclear. Therefore, further thorough and extensive analyses are necessary to illuminate these aspects in future studies. Isotopic labeling and metabolomics can be employed to track OCA in vivo and analyze its specific metabolites, which will provide a clearer understanding of the active form of OCA in the testis.

## Conclusions

Together, these findings suggest that OCA has the ability to efficiently revive sperm production and preserve the BTB structure in busulfan-treated mice by inhibiting oxidative stress and autophagy (Fig. 7). Consuming a balanced diet rich in OCA could improve male reproductive health. OCA shows promise as an innovative treatment option for male infertility, and incorporating OCA into regular dietary habits may enhance male fertility.

### Electronic supplementary material

Below is the link to the electronic supplementary material.

Supplementary Material 1

Supplementary Material 2

Supplementary Material 3

Supplementary Material 4

Supplementary Material 5

## Data Availability

No datasets were generated or analysed during the current study.

## Abbreviations

ART: Assisted reproductive technology
AZO: Azoospermia
BTB,: Blood-testis barrier
BW: Body weight
CAT: Catalase
EO: Extreme oligospermia
GDNF: Glial cell line-derived neurotrophic factor
HO1: Heme oxygenase 1
HTF: Human tubal fluid
LC3: Light chain 3
LGF: Liver growth factor
LPS: Lipopolysaccharide
NOA: Nonobstructive azoospermia
NQO1: NAD(P)H, quinone oxidoreductase 1
OCA: Octanoic acid
ROS: Reactive oxygen species
SOD: Superoxide dismutase
SQSTM1/P62: Sequestosome 1
TER: Transepithelial electrical resistance
