# Supplementary material for: Octanoic acid mitigates busulfan-induced blood-testis barrier damage by alleviating oxidative stress and autophagy
Source: Lipids Health Dis. 2024 Jun 11;23:180. doi: 10.1186/s12944-024-02157-2 (PMC11165768; doi:10.1186/s12944-024-02157-2)
Supplement: Supplementary file 1 — Supplementary Material 1 [file 12944_2024_2157_MOESM1_ESM.pdf]

iThenticate

638505165911702951
May 5, 2024
8655 words / 49405 characters

638505165911702951

Sources Overview

10%
OVERALL SIMILARITY

1	www.ncbi.nlm.nih.gov	INTERNET	1%
2	www.mdpi.com	INTERNET	<1%
3	www.frontiersin.org	INTERNET	<1%
4	respiratory-research.biomedcentral.com	INTERNET	<1%
5	Yuming Feng, Shanmeizi Zhao, Rong Ju, Jianbo Li et al. "The therapeutic effect and metabolic mechanism analysis of Guilingji on idiop...	CROSSREF	<1%
6	digital.csic.es	INTERNET	<1%
7	www.aging-us.com	INTERNET	<1%
8	Yongtao Liu, Yuntao Wang, Jianmin Zhang, Zhen Wang et al. "Regulation of survival, growth performance, and intestinal health of larg...	CROSSREF	<1%
9	ovarianresearch.biomedcentral.com	INTERNET	<1%
10	Shuxian Wang, Qiwei Chen, Yu Zhang, Feng Zheng et al. "Omega-3 polyunsaturated fatty acids alleviate hydrogen sulfide-induced bloo...	CROSSREF	<1%
11	www.spandidos-publications.com	INTERNET	<1%
12	Jianan Zhao, Minxin Wang, Yanan Wang, Jinyu Xu, Chenxu Ma, Yu Tang, QianQian Luo, Hongqin Zhang, Feibo Xu. "Endoplasmic reticul...	CROSSREF	<1%
13	www.jcancer.org	INTERNET	<1%
14	Jing-Bo Liu, Kai Chen, Zi-Fa Li, Zhen-Yong Wang, Lin Wang. "Glyphosate-induced gut microbiota dysbiosis facilitates male reproductive...	CROSSREF	<1%
15	Shanmeizi Zhao, Rujun Ma, Kadiliya Jueraitetibaike, Yao Xu et al. "ZDHHC17 participates in the pathogenesis of polycystic ovary syndr...	CROSSREF	<1%
16	Zhao, Jiaqiang, Lizhi Peng, Wenhua Zheng, Rikang Wang, Lei Zhang, Jian Yang, and Heru Chen. "Chemically Bonding of Amantadine wi...	CROSSREF	<1%
17	fjfsdata01prod.blob.core.windows.net	INTERNET	<1%

18 Jiabao Tang, Xiaohua Li, Wei Li, Chun Cao. "The effects of enteral nutrition supplemented with octanoic acid on LPS-induced intestinal ...
CROSSREF
<1%

19 evcna.com
INTERNET
<1%

20 link.springer.com
INTERNET
<1%

21 www.karger.com
INTERNET
<1%

22 LEE, Beom-Jun, Eun-Yong JUNG, Young-Won YUN, Jong-Koo KANG, In-Jeoung BAEK, Jung-Min YON, Yoon-Bok LEE, Heon-Soo SOHN, ...
CROSSREF
<1%

23 Tian-Hao Wang, Bi-Chen Zhao, Jin-Yang Li, Milton Talukder, Jin-Long Li. "Connexin-43 is a promising target for lycopene preventing pht...
CROSSREF
<1%

24 Jing-Bo Liu, Zi-Fa Li, Lu Lu, Zhen-Yong Wang, Lin Wang. "Glyphosate damages blood-testis barrier via NOX1-triggered oxidative stress ...
CROSSREF
<1%

25 Tayyaba Afsar, Suhail Razak, Janeen H. Trembley, Khushbukhat Khan et al. "Prevention of Testicular Damage by Indole Derivative MMI...
CROSSREF
<1%

26 Xiaoyu Zhang, Qin Xia, Rui Wei, Hongfei Song, Jiaqi Mi, Zhaoyu Lin, Yang Yang, Zijie Sun, Kang Zou. "Melatonin protects spermatogoni...
CROSSREF
<1%

27 assets.researchsquare.com
INTERNET
<1%

28 erj.ersjournals.com
INTERNET
<1%

29 www.amcmh.org
INTERNET
<1%

30 Bo Li, Xin He, Mengru Zhuang, Bowen Niu et al. "Melatonin Ameliorates Busulfan-Induced Spermatogonial Stem Cell Oxidative Apoptos...
CROSSREF
<1%

31 Jun Jing, Lei Ouyang, Hong Zhang, Kuan Liang et al. " Omega-3 polyunsaturated fatty acids and its metabolite rescue busulfan disrup...
CROSSREF
<1%

32 Miri Park, Hye-Bin Lee, Ha Ram Kim, Min-Cheol Kang, Duyun Jeong, Hee-Don Choi, Jung Sun Hong, Ho-Young Park. "Resistant starch-e...
CROSSREF
<1%

33 Shuyi Jiang, Ying Xu, Yunxia Fan, Ying Hu, Qiang Zhang, Wenhui Su. "Busulfan impairs blood–testis barrier and spermatogenesis by in...
CROSSREF
<1%

34 Yankang Cui, Chenkui Miao, Shouyong Liu, Jingyuan Tang et al. "Clusterin suppresses invasion and metastasis of testicular seminoma...
CROSSREF
<1%

35 journals.plos.org
INTERNET
<1%

36 www.oaepublish.com
INTERNET
<1%

37 www.science.gov
INTERNET
<1%

38 www2.mdpi.com
INTERNET
<1%

39 "Autophagy: Biology and Diseases", Springer Science and Business Media LLC, 2019
CROSSREF
<1%

40 Jihua Xu, Xiang Zhang, Xiaohong Sun, Qiang Lv, Yugang Zhang. "Red-Fleshed Apple Anthocyanin Extracts Attenuate Male Reproductiv...
CROSSREF
<1%

41 AMOURA M. ABOU-EL-NAGA, SHAKER A. MOUSA, FAYEZ ALTHOBAITI, EMAN FAYAD, ENGY S. FAHIM. "Ameliorative effects of melatoni...
CROSSREF
<1%

42 Faridah Hanim Shakirin, Azrina Azlan, Amin Ismail, Zulkhairi Amom, Lau Cheng Yuon. " Protective Effect of Pulp Oil Extracted from Miq.... <1%
CROSSREF

43 Marzieh Zeinvand-Lorestani, Samaneh Karimi, Layasadat Khorsandi. "Quercetin ameliorates cytotoxic effects of zinc oxide nanopartic... <1%
CROSSREF

44 Rujun Ma, Hongru Li, Yu Zhang, Ying Lin, Xuhua Qiu, Min Xie, Bing Yao. "The toxic effects and possible mechanisms of Brusatol on mou... <1%
CROSSREF

45 Yuan Yang, Guoqing Fu, Xueyi Zhao, Xiaoping Wu et al. "Perfluorooctanoic acid induces tight junction injury of Sertoli cells by blocking ... <1%
CROSSREF

Excluded search repositories:

- None

Excluded from document:

- Bibliography

Excluded sources:

- None

Excluded preprints

- None

Octanoic acid mitigates busulfan-induced blood–testis barrier damage by alleviating oxidative stress and autophagy

Chun Cao[a,1], Hong Zhang[b,1], Zhaowanyue He[b,1], Kemei Zhang[c], Zhang Qian[b], Jiaming Shen[b], Lu Zheng[b], Mengqi Xue[c], Shanshan Sun[d], Chuwei Li[b], Wei Zhao[b], Jun Jing[c], Rujun Ma[a,b], Xie Ge[a,b,*], Bing Yao[a,b,c,d,*].

Author affiliations

a Department of Reproductive Medicine, Affiliated Jinling Hospital, The First School of Clinical Medicine, Southern Medical University, Nanjing, 210002, China

b Center of Reproductive Medicine, Jinling Hospital, Affiliated Hospital of Medical School, Nanjing University, Nanjing, Jiangsu, 210002, China

c Reproductive Medical Center, Jinling Hospital Department, Nanjing Medical University, Nanjing, Jiangsu, 210002, China

d College of Life Sciences, Nanjing Normal University, Nanjing, Jiangsu, 210023, China

1 These authors contributed equally: Chun Cao, Hong Zhang, Zhaowanyue He.

*** Correspondence:**

Bing Yao, PhD

Department of Reproductive Medicine, Affiliated Jinling Hospital, The First School of Clinical Medicine, Southern Medical University, Nanjing, China. 305 Zhongshan East Road.

E-mail: yaobing@nju.edu.cn

Tel numbers: +86 13815853296

Xie Ge, PhD

Center of Reproductive Medicine, Jinling Hospital, Affiliated Hospital of Medical School, Nanjing University, Nanjing, Jiangsu, China. 305 Zhongshan East Road.

E-mail: gexie85@163.com

Tel numbers: +86 18362952185

Abstract

Background: The management of male infertility continues to encounter an array of challenges and constraints, necessitating an in-depth exploration of novel therapeutic targets to enhance its efficacy. As an eight-carbon medium-chain fatty acid, octanoic acid (OCA) shows promise for improving health, yet its impact on spermatogenesis remains inadequately researched.

Methods: Mass spectrometry was performed to determine the fatty acid content and screen for a pivotal lipid component in the serum of patients with severe spermatogenesis disorders. The sperm quality was examined, and histopathological analysis and biotin tracer tests were performed to assess spermatogenesis function and the integrity of the blood-testis barrier (BTB) *in vivo*. To investigate the effects of OCA administration on Sertoli cell dysfunction, cell-based in vitro experiments were carried out. This research aimed to uncover the mechanism by which OCA may influence the functioning of Sertoli cells.

Results: A pronounced reduction in OCA content was observed in the serum of patients with severe spermatogenesis disorders, indicating that OCA deficiency is related to spermatogenic disorders. The protective effect of OCA on reproduction was tested in a mouse model with spermatogenic disorder induced by busulfan (30 mg/kg BW). The mice in the study were separated into distinct groups and administered varying amounts of OCA, specifically at doses of 32, 64, 128, and 256 mg/kg. After evaluating sperm parameters, the most effective dose was determined to be 32 mg/kg. *In vivo* experiments showed that treatment with OCA significantly improved sperm quality, testicular histopathology and BTB integrity, which were damaged by busulfan. Moreover, OCA intervention reduced busulfan-induced oxidative stress and autophagy in mouse testes. *In vitro*, OCA pretreatment (100 μM) significantly ameliorated Sertoli cell dysfunction by alleviating busulfan (800 μM)-induced oxidative stress and autophagy. Moreover, rapamycin (5 mM)-induced autophagy led to Sertoli cell barrier dysfunction, and OCA administration exerted a protective effect by alleviating autophagy.

Conclusions: This study demonstrated that OCA administration suppressed oxidative stress and autophagy to alleviate busulfan-induced BTB damage. These findings provide a deeper understanding of the toxicology of busulfan and a promising avenue for the development of novel OCA-based therapies for male infertility.

Keywords: Medium-chain fatty acids, octanoic acid, busulfan, spermatogenesis disorder, blood–testis barrier, autophagy, oxidative stress, male infertility.

Introduction

Infertility has emerged as a significant global health concern, and male factors contribute to at least

50% of cases [1,2]. Spermatogenesis disorders represent one of the most severe and intricate manifestations of male infertility. In recent decades, significant progress has been made by scientists toward comprehending the underlying causes and mechanisms associated with spermatogenesis disorders. Various adverse factors, including genetic mutations, environmental influences, and exposure to chemotherapy drugs, can contribute to spermatogenesis disorders [2–4]. Busulfan, a commonly used alkylating agent with nonspecific effects, is frequently given as a preparatory regimen before allogeneic hematopoietic stem cell transplantation. Studies have shown that it can lead to disruptions in male sperm production [5,6]. Animal models induced by Busulfan for studying spermatogenesis disorders are widely used to explore the mechanisms of new medications and therapies for male infertility [7].

Despite the availability of assisted reproductive technology (ART) as a viable approach to address specific fertility challenges, the universal applicability of these technologies remains limited [8]. Furthermore, despite encouraging outcomes observed following the administration of recently identified compounds like liver growth factor (LGF), melatonin, glial cell line-derived neurotrophic factor (GDNF), and others, rigorous investigations will be paramount to ascertain their long-term efficacy and safety, particularly in human subjects [9–11]. Consequently, there is an imperative need to discover novel therapeutic targets and enhance the efficacy of existing interventions for male infertility caused by spermatogenesis disorders.

Fatty acids are a category of organic compounds characterized by extended hydrocarbon chains. These biomolecules hold paramount significance within the body due to their roles in orchestrating multifaceted pathways, including contributing to the structural integrity of cellular membranes, modulating metabolic pathways, and actively participating in intricate physiological processes [12]. Recent investigations have predominantly focused on elucidating the beneficial effects of unsaturated fatty acids in the context of male reproductive health. Consuming omega-3 polyunsaturated fatty acids is thought to be important in preserving hormonal equilibrium, promoting the typical development of reproductive cells, and sustaining the structural integrity and motility of sperm [13–15]. Despite this, unsaturated fatty acids are inherently more prone to

oxidation and heat-induced breakdown compared to saturated fats, which can make them more challenging to acquire [16]. Therefore, a more comprehensive exploration is warranted to identify a fatty acid that is easily accessible and beneficial to male reproductive function.

Caprylic acid (OCA) is an eight-carbon medium-chain saturated fatty acid commonly found in coconut oil and dairy products [17,18]. Several studies in humans have demonstrated the potential health benefits of OCA, including its ability to improve lipid metabolism and suppress inflammatory responses [19]. Additionally, OCA treatment has been shown to alleviate H_2O_2-induced oxidative stress, which can improve the development of liver disease [20]. Furthermore, OCA supplementation has been found to prevent lipopolysaccharide (LPS)-induced acute liver injury by upregulating autophagy [21]. Although OCA supplementation is potentially beneficial to human health, whether OCA plays a role in spermatogenesis and male reproductive health remains unclear.

Hypothesized from these inquiries, it can be argued that OCA has the potential to reduce busulfan-induced damage in spermatogenesis, leading to improvements in male fertility. To verify this hypothesis, the potential therapeutic effects of OCA supplementation were firstly investigated using a busulfan-induced mouse model of spermatogenesis disorder and then determined the sperm quality and blood-testis barrier (BTB) integrity. The findings indicate that supplementation of OCA might potentially benefit spermatogenesis disorders and BTB damage, highlighting its potential as a treatment for male infertility. Furthermore, the therapeutic effect of OCA can be attributed to its ability to alleviate busulfan-induced oxidative stress and autophagy. The findings presented in this study provide valuable insights into how busulfan contributes to reproductive toxicity and the potential for developing more effective treatments for male infertility.

Materials and methods

Analysis of human serum fatty acids and sperm quality

During the period from September 2017 to January 2020, serum specimens were collected from thirty subjects experiencing spermatogenesis abnormalities. This group consisted of 24 patients diagnosed with nonobstructive azoospermia (NOA) and 6 with extreme oligospermia (EO).

Additionally, 30 individuals without any known fertility issues, who were in the process of receiving standard semen assessments at the medical laboratory, served as the control group (Supplementary Table S1). The participants in this study were between the ages of 20 and 43, and blood samples were collected between 8:00 and 11:00 am. Subsequently, centrifugation at 1800×g for 10 min at room temperature was used to separate the serum. In previous research [22], the evaluation of serum levels of free fatty acids was outlined. Shanghai Applied Protein Technology Co. Ltd utilized GC/MS to measure 39 medium- and long-chain free fatty acids (C6–C24). Sperm quality was assessed with the WLJY-9000 computer-assisted system (WLJY-9000, WeiLi, Beijing, China). Collection of clinical samples was conducted with informed consent and authorized by the Research Ethics Committee of Jinling Hospital.

Materials and reagents

Thermo Fisher Scientific (Massachusetts, USA) provided the EZ-Link Sulfo-NHS-LC-Biotin (21335) Octanoic acid (OCA) (C2875), busulfan (55-98-1), and Alexa Fluor 488-conjugated streptavidin (S32354). Beyotime Biotechnology (Shanghai, China) provided the Protease inhibitor cocktail (P1005), phenylmethylsulfonyl fluoride (PMSF) (ST507), Rapamycin (S1842), Ad-GFP-LC3B (C3006), the Total Superoxide Dismutase (SOD) Assay Kit (S0101M), 2′,7′-dichlorofluorescin diacetate (DCFH-DA) (S0033 M) and the Lipid Peroxidation Malondialdehyde (MDA) Assay Kit (S0131M). Nanjing KeyGen Biotechnology (Jiangsu, China) provided the CCK-8 assay kit. Vazyme Biotechnology (Nanjing, China) provided the HiScript III RT SuperMix for qPCR (R323) and AceQ qPCR SYBR Green Master Mix (Q121).

Animals and experimental design

The research utilized 60 male C57BL/6 mice, aged 4 weeks, obtained from Beijing Vital River Laboratory Animal Technology Co. Ltd (Beijing, China), for the purpose of conducting *in vivo* experiments. Fig. 2A depicts the experimental layout. Following a 7-day acclimatization period, the mice were distributed randomly into six categories: control, busulfan alone, and four OCA therapy sets at doses of 32, 64, 128, and 256 mg/kg body weight (BW), with each group containing 10 mice. The control group received Coil oil orally for three weeks and then a single intraperitoneal injection of DMSO. The busulfan alone group was given Coil oil orally and busulfan (30 mg/kg BW) injection intraperitoneally for 3 weeks. The OCA + busulfan group received OCA in Coil oil orally,

followed by a single intraperitoneal injection of busulfan (30 mg/kg). OCA treatment continued for five weeks, after which the mice were euthanized for testis sample collection and subsequent analysis.

The initial busulfan concentration was 30 mg/mL in DMSO, which was thoroughly mixed with an equivalent volume of PBS in an ultrasonic device at 37 °C to reduce its toxicity. The mice were first euthanized using ether and then sacrificed through cervical dislocation to collect testis and epididymis samples. Cervical dislocation post-anesthesia is deemed a humane euthanasia method as outlined in the Guide for the Care and Use of Laboratory Animals (Eighth Edition) by the Institutional Animal Care & Use Committee. The experimental protocols were conducted in adherence to the standards established by the National Laboratory Animal Care and Use Research Committee.

TM4 cell treatment

To find the best treatment concentrations of OCA and busulfan, busulfan was dissolved in DMSO, while OCA was dissolved in alcohol and both were diluted in culture medium. Equal amounts of DMSO and alcohol were added to the control, busulfan, and OCA groups, at a final concentration of less than 0.1%. Based on previous studies [23], TM4 cells were either pretreated with OCA (100 μM) for 2 h or exposed to busulfan (800 μM) for 24 h. The optimal OCA amount was established through the CCK-8 assay. Furthermore, in the rapamycin intervention experiments, TM4 cells were pre-exposed to OCA for 2 h before being subjected to rapamycin (5mM), as confirmed by the CCK-8 assay.

Sperm quality analysis

The method for evaluating sperm quality was previously described [22]. The epididymides were recently transferred into HTF medium and were sliced into small fragments with ophthalmic scissors. Following an incubation period at 37 °C for 5 min, a hemocytometer (Qiujing, located in Shanghai, China) was used to observe 10 μL of the sperm suspension with the assistance of a light microscope (Olympus, Tokyo, Japan). The numbers of forward-moving sperm, non-forward-moving sperm, and immobile sperm were counted separately.

Histology analysis of the testis and epididymis

The samples from the testis and epididymis were fixed through immersing in Bouin's solution for

24 h, followed by dehydration using ethanol of varying concentrations. Subsequently, they were embedded in paraffin for the purpose of sectioning. Sections of tissue, 5 μm thick, were then stained using hematoxylin and eosin (HE) before being captured and digitized under an optical microscope.

BTB integrity analysis

The evaluation of BTB integrity was carried out with a biotin tracer, as detailed in previous studies [24,25]. After receiving a 5-week busulfan treatment, three mice were chosen at random from each experimental group for additional analysis. Then mice were injected intraperitoneally with 1% sodium pentobarbital (5 mL/kg) to anesthetize. Incisions were created in the lower abdomen of the mice to expose their testes. Subsequently, 20 μL of freshly prepared EZ-Link Sulfo-NHS-LC-Biotin in PBS with 1 mM $CaCl_2$ was injected into the stroma of the upper, middle, and lower testicular regions. The testes were extracted after 30 min of diffusion and promptly frozen in liquid nitrogen for cryosectioning. The slices, 10 μm in thickness, were immersed in 4% paraformaldehyde for 10 min, then treated with Alexa Fluor 488-labeled streptavidin for 1 h at room temperature. The specimens were then stained with DAPI and examined using a fluorescence microscope to visualize the seminiferous tubules. The assessment of BTB damage was determined using the subsequent equation: A total of 50-60 round or oval-shaped cross-sections of the seminiferous tubules were randomly examined in each group. For oval-shaped tubules, the D_{biotin} was computed as the average of the long and short axes of the tubule.

$$\text{The extent of BTB damage} = \frac{D_{biotin}}{D_{radius}} 100\%$$

(D_{biotin}: the diffusion distance of biotin; D_{radius}: the radius of the tube)

Western blot

Testicular samples and cells were processed by undergoing different treatments and being lysed in RIPA buffer. The proteins were extracted by sonication on ice, followed by centrifugation at high speed and low temperature. Following the quantification of protein concentrations, protein (20 μg) was then resolved using SDS–PAGE, and later transferred to PVDF membranes (Millipore, Massachusetts, USA). Following the blocking process with BSA, both primary and secondary antibodies were added to the membranes. The primary antibodies were used in the present study: A ZO-1 rabbit pAb (21773-1-AP, ProteinTech, Wuhan, China), a Claudin11 rabbit pAb (AF5364, Affinity, Ohio, USA), a Claudin5 rabbit pAb (AF5216, Affinity), a Occludin rabbit pAb (13409-1-

AP, ProteinTech), a HO1/HMOX1 rabbit pAb (10701-1-AP, ProteinTech), a NQO1 rabbit pAb (11451-1-AP, ProteinTech), a SQSTM1/P62 rabbit mAb (ab109012, Abcam, Shanghai, China), a LC3B rabbit mAb (ab192890, Abcam). The visualization of the target protein bands was carried out using the Chemiluminescent Imaging System. The protein bands were subsequently quantified using ImageJ.

Analysis using real-time PCR

Gene expression levels related to oxidative stress were measured through RT-PCR analysis in this investigation. After isolating total RNA from the testes using a Total RNA Purification Kit (082001, BEI-BEI Biotech, Zhengzhou, China), cDNA synthesis was conducted utilizing HiScript III RT SuperMix for qPCR following evaluation of RNA concentration and integrity. The quantitative analysis was carried out on a Roche LightCycler 96 Real-time PCR machine (Roche Diagnostics, Basel, Switzerland) using qPCR SYBR Green Master Mix. The reference gene β-actin served as the Uniform labeling, and gene expression values were calculated using the $2^{-\Delta\Delta Cq}$ formula [26]. Please see Table S3 for the primer sequences applied in the study.

Measurement of SOD and MDA

In brief, the tissue samples were processed by homogenizing them and then centrifuging to extract protein samples. Following the prescribed protocol, the activity of SOD and the concentration of MDA were measured using specific assay kits.

Intracellular ROS quantification

The levels of ROS within the cells were quantified utilizing a DCFH-DA fluorescent probe in adherence to the guidelines provided by the manufacturer. Following treatment, 10 μM DCFH-DA solution in medium devoid of FBS was administered to each corresponding well. Afterward, the cells were incubated in a lightless setting at 37 °C for 20 min prior to being rinsed twice with medium devoid of FBS. The brightness of the fluorescence was captured with a fluorescence microscope.

Transepithelial electrical resistance (TER) evaluation

To evaluate cell barrier performance in a controlled environment, TER was measured daily in three specific regions of the samples using a Milli-cell ERS apparatus (Millipore). Sertoli cells were initially inoculated in MilliCell Hanging Cell Culture Inserts (PET 0.4 μm, Millipore) at a

concentration of 0.5 × 10^6 cells/cm^2 and allowed to develop for three days to establish cellular barriers. Post-treatment, the Millicell Electrical Resistance System (Millipore) was used to capture the TER readings. The calculation of the TER value was determined by the following formula: TER ($\Omega \cdot cm^2$) = (resistance from treatment (Ω) - initial resistance (Ω)) × surface area of membrane (cm^2).

Statistical analysis

The data illustrated in this investigation were sourced from a minimum of three distinct *in vivo* specimens and three separate *in vitro* trials. The outcomes were visualized utilizing GraphPad Prism 7 (GraphPad Software, California, USA) and reported as the means ± standard deviations (SD). Statistical significance across various groups was scrutinized utilizing SPSS 19.0 software (SPSS, Illinois, USA) through independent t-tests and one-way analyses of variance (ANOVAs) succeeded by post hoc assessments of least significant divergence (matching variances) or Games-Howell (mismatching variances). A significance level of $P < 0.05$ was used to determine statistical relevance.

Results

Serum OCA levels are significantly lower in azoospermic patients

To identify the potential fatty acids that are potentially beneficial to male reproductive function, a comprehensive clinical analysis was performed. This analysis involved a comparative examination of the fatty acid compositions (C6-C24) of serum samples from two cohorts—30 healthy individuals and 30 patients—diagnosed as azoospermia (AZO), including NOA and EO. Through the application of GC/MS for quantifying the serum concentrations of 39 medium- and long-chain fatty acids, notable modifications were identified in the fatty acid composition within the serum of individuals compared with controls (Fig. 1A), showcasing a marked reduction in the levels of C6 and C8 fatty acids with statistical significance ($P < 0.0001$) (Fig. 1B-C). Furthermore, a greater decrease in levels of octanoic acid (OCA, C6) was noted in the study (Fig. 1C), indicating that supplementing with OCA could potentially improve spermatogenesis. At the same time, there was a notable reduction ($P < 0.0001$) in the production of inhibin B (INHB) (Fig. 1E), a reproductive hormone released by Sertoli cells, in the plasma of these individuals, whose sperm count exhibited a corresponding decline ($P < 0.0001$) (Fig. 1D). These findings from the medical evaluation suggest a potential link between OCA deficiency and impaired Sertoli cell function.

OCA improves spermatogenesis disorders in busulfan-treated mice

To explore whether OCA supplementation can ameliorate spermatogenesis disorders and Sertoli cell dysfunction, a mouse model with impaired spermatogenesis was induced using a single intraperitoneal dose of busulfan. Subsequently, OCA was given to these mice orally, following the procedure depicted in Fig. 2A. Previous investigations have demonstrated that the highest dose of OCA (128 mg/kg) did not cause dose-limiting toxicity, and the most common adverse event observed was mild abdominal discomfort, indicating that administration of OCA via gavage is safe and reliable [27]. Therefore, four concentrations of OCA (32, 64, 128, and 256 mg/kg BW) were used to investigate the potential therapeutic effects of OCA via gavage (Fig. 2A).

Compared to the control, the OCA-receiving group exhibited a notable reduction in sperm concentration ($P < 0.0001$), forward movement of sperm ($P < 0.0001$), weight of the testis ($P < 0.0001$) and epididymis ($P < 0.01$), and the coefficients of organs including the testis ($P < 0.0001$) and epididymis ($P < 0.05$) (Fig. 2B-E, Fig. S1A-B), indicating the induction of spermatogenesis disorder by busulfan. However, mice administered each of the four doses of OCA (32, 64, 128, or 256 mg/kg BW) displayed significantly elevated sperm concentrations, sperm progressive motility, testis and epididymis weights, and organ coefficients of the testis and epididymis relative to mice solely receiving busulfan (Fig. 2B-E, Fig. S1A-B), suggesting the successful amelioration of busulfan-induced spermatogenesis disorder through OCA administration. Furthermore, histological analysis of epididymal tubules using hematoxylin and eosin (HE) staining revealed a significant decrease in sperm density in the epididymal cauda of the mice in the busulfan group, which was also restored by OCA supplementation (Fig. S1C). Based on these results, it can be inferred that a clear therapeutic effect existed in the groups treated with OCA, and a better effect was observed at the lower concentration (32 mg/kg). Therefore, most of the following experiments were performed using this dose.

To determine the protective effects of OCA supplementation on testicular health, the histopathology of the testes was evaluated using HE staining. Fig. 2F illustrates testicular morphology of the busulfan group experienced noticeable changes in the seminiferous tubules, including atrophy,

vacuolation, and loss of germ cells. Conversely, the group supplemented with OCA showed well-organized seminiferous tubules and the reemergence of germ cells. Additionally, the dimensions of seminiferous tubules and the thickness of seminiferous epitheliums were both measured. A notable decline ($P < 0.001$) was noted in these variables in the busulfan-treated group, whereas a rise ($P < 0.001$) was seen in the group treated with OCA (Fig. 2G-H). Furthermore, there was a notable increase in abnormal tubules (including empty and partial types) after busulfan administration, but this proportion was notably reduced in the OCA-administered group (Fig. 2I). These findings suggest that OCA supplementation can expedite the regeneration of spermatogenesis impaired by busulfan treatment in mice.

OCA supplementation restores busulfan-disrupted BTB integrity in mouse testes

To validate whether OCA supplementation could alleviate the effects of busulfan toxicity on spermatogenesis by restoring Sertoli cell function, a semiquantitative was employed *in vivo* assay in which biotin was used as an indicator. Demonstrated in Fig. 3A-B, a fully functional BTB obstructed the entry of biotin into the seminiferous lumen. This led to detection of streptavidin-488 fluorescence solely within the testicular interstitium and basal membranes of the control group mice. In contrast, injection of busulfan resulted in a greater permeation distance of biotin by approximately 60% ($P < 0.0001$), while supplementation with OCA reduced the leakage of biotin by approximately 17% ($P < 0.001$), indicating that OCA treatment restored BTB integrity. Busulfan administration also significantly reduced the levels of BTB-related connexins: ZO-1, occludin, claudin5 and claudin11 ($P < 0.01$, $P < 0.05$, $P < 0.01$, $P < 0.05$, respectively) (Fig. 3C-H). Conversely, treatment with OCA led to a noticeable rise in the expression of these proteins ($P < 0.01$, $P < 0.05$, $P < 0.05$, $P < 0.01$) (Fig. 3C-H). These results indicate that OCA may hold promise in ameliorating Sertoli cell dysfunction observed in mice treated with busulfan.

OCA alleviates oxidative stress and autophagy induced by busulfan in mouse testes

Previous studies have demonstrated that OCA can increase antioxidant capacity and regulate autophagy to benefit the functions of biological systems [21,28,29]. To explore whether OCA could alleviate oxidative stress levels to restore Sertoli cell dysfunction *in vivo*, testicular SOD activity

and MDA levels were assessed, which were significantly increased ($P < 0.0001$) in the busulfan-treated group and decreased ($P < 0.01$) in mice after OCA treatment (Fig. 4A-B). Additional studies were carried out to assess oxidative stress-related genes expression in testes. The mRNA levels of *Nqo1*, *Ho1*, *Cat*, and *Sod3* were significantly increased ($P < 0.001$) by busulfan treatment but decreased ($P < 0.001$) after OCA supplementation (Fig. 4C-F). Additionally, compared with busulfan treatment, OCA treatment significantly decreased the protein levels of NQO1 and HO1 ($P < 0.05$, $P < 0.001$) (Fig. 4G-I). In addition, the levels of autophagy-associated proteins LC3B and P62, representing autophagosome formation and breakdown, were assessed. Immunofluorescence staining demonstrated the presence of both LC3 and P62 in Sertoli and spermatogenic cell layers in the control group (Fig. 4G). After being exposed to busulfan, there was a marked rise in the LC3-II/LC3-I ratio ($P < 0.05$), along with a significant drop in P62 levels ($P < 0.001$) (Fig. 4K-M), indicating busulfan's ability to trigger autophagy and enhance autophagic breakdown in the testes. Conversely, supplementation with OCA resulted in contrasting trends in the LC3-II/LC3-I ratio and P62 expression levels compared to the busulfan group (Fig. 4K-M). The findings imply that OCA could mitigate the oxidative stress and autophagy caused by busulfan in the testes, thereby promoting the restoration of spermatogenesis and Sertoli cell function.

OCA supplementation alleviates busulfan-induced oxidative stress and autophagy in TM4 Sertoli cells

The study employed the Sertoli cell line TM4 to investigate the efficacy of OCA supplementation in reversing dysfunction in Sertoli cells by shielding them from oxidative stress and hindering autophagy *in vitro*. Cytotoxicity was initiated by subjecting TM4 cells to 800 μM busulfan, followed by treatment with varying therapeutic concentrations of OCA (100, 200, or 400 μM). Cell viability was assessed through a CCK-8 assay, as illustrated in Figure 5A. Interestingly, the administration of 100 μM OCA led to a noteworthy enhancement ($P < 0.05$) in cell viability. Therefore, subsequent therapeutic experiments were conducted *in vitro* using this dose. Transepithelial electrical resistance (TER) assays revealed that compared with busulfan treatment, OCA treatment notably increased ($P < 0.01$) electrical resistance (Fig. 5B), indicating that OCA treatment protected against cell barrier damage caused by busulfan. Additionally, the OCA therapy effectively reduced the decrease in

BTB-related proteins (ZO-1 and Occludin) triggered by busulfan (Fig. 5C and 5E). Moreover, the levels of HO1, NQO1, P62 and LC3B proteins were analyzed in TM4 Sertoli cells (Fig. 5D-E). OCA intervention alleviated the reduction in P62 protein levels and the elevation in HO1 and NQO1 levels, as well as the LC3II/LC3I ratio compared to those in the busulfan-exposed group (Fig. 5D-E). Additionally, compared to busulfan treatment alone, OCA treatment significantly decreased the intracellular ROS levels and cytoplasmic GFP-positive puncta (Fig. 5F-G). This suggests that OCA treatment effectively mitigated the oxidative stress and autophagy triggered by busulfan *in vitro*, aligning with the outcomes of the conducted *in vivo* trials. These results suggest that OCA can improve busulfan-induced Sertoli cell dysfunction through the suppression of oxidative stress and autophagy.

OCA treatment alleviates TM4 cell damage by suppressing autophagy

To validate the role of autophagy in Sertoli cell barrier integrity and the therapeutic effect of OCA, rapamycin, an autophagy activator, was used to induce autophagy *in vitro*. Based on the results of the CCK-8 assay (Fig. 6A-B), 5 μM rapamycin and 100 μM OCA were employed in *in vitro* intervention assay. In vitro experiments showed that rapamycin, similar to the effect of busulfan, had a significant negative effect on Sertoli cell barrier function (Fig. 6C-E), indicating that activating autophagy can disrupt this important barrier. Additionally, in the OCA-treated group, there was a notable increase in electrical resistance and elevated protein levels of ZO-1 and Occludin compared to those in cells treated only with rapamycin. These findings strongly indicate that OCA plays a pivotal role in restoring Sertoli cell barrier function. Furthermore, compared with rapamycin treatment, supplementation with OCA induced a significant decrease in GFP-LC3 fluorescence and the LC3II/LC3I ratio (Fig. 6F-G) and an increase in P62 expression (Fig. 6H-I). These findings indicate that OCA treatment can rescue Sertoli cell barrier damage by inhibiting autophagy.

Discussion

In the course of this study, it was observed that individuals suffering from severe dyszoospermia exhibited a decrease in serum levels of OCA. This observation implies that supplementation with OCA could ameliorate spermatogenesis disorders (Fig. 1). In this study, it could be found that OCA

supplementation rescues spermatogenesis by reducing oxidative stress and autophagy levels in Sertoli cells.

Prior research has indicated that busulfan has the ability to inhibit spermatogenesis by harming germ cells directly or interfering with the BTB, resulting in male infertility [25,31]. A recent investigation by Zhao et al. suggested that Sertoli cells are vital in the progression of dyszoospermia [32]. An essential role of Sertoli cells is establishing the BTB, creating a specific environment for germ cell growth that is shielded from potentially harmful compounds and immune reactions towards sperm antigens [33]. In this study, it was demonstrated that OCA supplementation restored busulfan-induced damage to BTB integrity (Fig. 3). In addition it was noted the serum INHB levels were decreased in patients with clinical spermatogenic dysfunction (Fig. 1E). Additional studies have demonstrated that busulfan has adverse effects on Sertoli cells [34]. Given the above findings, the impact of OCA was focused on Sertoli cell. This research notably displayed the advantageous impacts of OCA on Sertoli cells and the BTB.

Oxidative stress is a significant factor in male infertility, with an imbalance of ROS often cited as a main cause [35]. This imbalance has also been identified as a key contributor to busulfan-induced reproductive toxicity [36]. Accumulating *in vitro* and *in vivo* evidence has elucidated the multifaceted mechanisms by which OCA ameliorates various diseases, notably through the alleviation of oxidative stress [29,37]. In this study, it was found that OCA treatment effectively improved busulfan-induced oxidative stress in the testes. Additionally, *in vitro* study using TM4 cells confirmed the ability of OCA to mitigate busulfan-induced oxidative stress.

Autophagy, a lysosomal catabolic mechanism present in all eukaryotes, is essential for maintaining a balanced cellular environment by degrading proteins and organelles [38]. This process is intimately linked to male reproduction [39], and dysregulation of autophagy in Sertoli cells has been linked to male infertility and testicular dysfunction, including BTB destruction [40]. Modulating autophagy is being explored as a promising therapeutic approach for Sertoli cell dysfunction. Recent studies have shown that enteral nutrition rich in OCA can prevent acute liver injury induced by LPS

through the upregulation of autophagy [21]. Therefore, autophagy may represent a potential mechanism by which OCA can improve Sertoli cell dysfunction. Furthermore, supplementation with OCA has been found to alleviate autophagy induced by busulfan or an autophagy agonist (Figs. 4, 5 and 6), indicating its potential protective effects against BTB damage. These findings highlight the therapeutic value of targeting autophagy for treating male infertility.

Contrary to the results presented in this study, Wei and colleagues observed that busulfan suppresses autophagy by halting the breakdown of autophagosomes, leading to the impairment of spermatogonial cells [41]. However, this finding does not contradict this study, as the effect of autophagy on male fertility is twofold [42]. Optimal autophagy can shield cells effectively from unfavorable environmental conditions, such as hypoxia, lack of nutrients, oxidative stress, or the buildup of misfolded proteins. In times of stress, the upregulation of autophagy enables the cell to acclimate to changing surroundings and ensure its survival [43]. Nonetheless, excessive autophagy can result in the excessive consumption of proteins, harm to cell organelles, and impairment of cellular function, ultimately leading to defects in spermatogenesis and damages in the blood-testis barrier (BTB) [44–46]. This discovery implies that the status of autophagy may differ under varying treatments. In conjunction with the current study, these findings underscore the importance of autophagy in busulfan toxicology, as well as the protective influence of OCA by regulating autophagy.

It is noteworthy that in this investigation, no dose-response correlation was found with OCA, as mice receiving the lowest experimental dosage (32 mg/kg) showed marked enhancements. Remarkably, adding high doses of OCA has been found to produce adverse impacts on the growth of blastocysts, rates of hatching, speed of development, and overall cell count [30]. Therefore, it can be speculated that the administration of OCA is a double-edged sword and that high doses of OCA may have adverse effects on spermatogenesis development in mice, indicating that the amount of octanoic acid intake should be moderate. When OCA is used for the treatment of dyszoospermia, its dose should be precisely controlled to prevent its side effects.

Because of its reduced molecular size, OCA can be absorbed straight from the intestines, skipping the lymphatic system and entering the liver through the portal vein directly [47]. Studies have demonstrated that the concentration of OCA in plasma increases after oral OCA administration [27]. According to a radiolabeled fatty acid tracer study, OCA can be rapidly transported into the central nervous system following oral administration [48]. These findings indicate that OCA can possibly reach testes through the blood circulation and exert its effects in the spermatogenesis microenvironment. The *in vitro* experiments confirmed the antioxidative and anti-autophagic effects of OCA on Sertoli cells. This, in turn, leads to an improvement in Sertoli cell barrier function, consistent with the outcomes of *in vivo* gavage studies. In addition, OCA can also be metabolized into small molecule substances (e.g., ketone bodies) or synthesized into long-chain fatty acids to exert their biological functions [49,50]. With respect to the functions of its metabolites, further investigations following a comprehensive analysis of the fatty acid composition of the local testicular milieu will improve the understanding of how OCA works *in vivo*.

Strengths and limitations

The research conducted focused on examining the effects of OCA on spermatogenesis disorder in mice induced by busulfan, specifically looking at how OCA can enhance spermatogenesis by safeguarding Sertoli cells. The results showed that OCA can reduce oxidative stress and regulate autophagy in Sertoli cells. This study is the inaugural attempt to investigate the therapeutic mechanism of OCA in addressing male infertility. It is hoped that OCA could be utilized as a promising dietary intervention to enhance spermatogenesis in individuals with male infertility issues.

However, in this research, the impacts of OCA were predominantly evaluated on Sertoli cells, while the effects of OCA on germ cells must not be disregarded and warrant future examination. If circumstances allow, single-cell omics may be utilized to examine the impacts of OCA on all cell types within the testis. Despite the results of this research supporting the idea that OCA could enhance spermatogenesis by mitigating oxidative stress and autophagy in Sertoli cells, the precise bioactive substances and metabolic components responsible for these therapeutic effects are still unclear. Therefore, further thorough and extensive analyses are necessary to illuminate these aspects

in forthcoming studies. Isotopic labeling and metabolomics can be employed to track OCA in vivo and analyze its specific metabolites, which will provide a clearer understanding of the active form of OCA in the testis.

Conclusions

Together, these findings suggest that OCA has the ability to efficiently revive sperm production and preserve BTB structure in busulfan-treated mice by inhibiting of oxidative stress and autophagy. Consuming a balanced diet rich in OCA could potentially improve male reproductive health. OCA shows promise as an innovative treatment option for male infertility, and incorporating OCA into regular dietary habits may enhance male fertility.

Abbreviations

ART	assisted reproductive technology
AZO	azoospermia
BTB,	blood-testis barrier
BW	body weight
CAT	catalase
EO	extreme oligospermia
GDNF	glial cell line-derived neurotrophic factor
HO1	heme oxygenase-1
HTF	human tubal fluid
LC3	light chain 3
LGF	liver growth factor
LPS	lipopolysaccharide
MDA	malondialdehyde
NOA	nonobstructive azoospermia
NQO1	NAD(P)H, quinone oxidoreductase 1
OCA	octanoic acid
ROS	reactive oxygen species
SOD	superoxide dismutase
SQSTM1/P62	sequestosome 1

Ethics approval

Ethical guidelines established by relevant agencies govern all human experimentation and ensure that full consent is obtained from patients before experiments are conducted. All animal experiments were systematically evaluated and sanctioned by the Animal Protection and Use Committee of

Jinling Hospital.

Funding

Financial assistance for this investigation was provided by the National Natural Science Foundation of China (U22A20277, 81901547, and 81971373) and the Jiangsu Provincial Medical Key Discipline Cultivation Unit (JSDW202215).

Author contributions

Chun Cao, Hong Zhang and Zhaowanyue He: conducted the study, retrieved and analyzed the data, and drafted the article. Kemei Zhang, Zhang Qian, Jiaming Shen, and Lu Zheng conducted the investigations. Mengqi Xue, Shanshan Sun and Chuwei Li examined the data. Wei Zhao, Jun Jing and Rujun Ma helped perform the analysis. Xie Ge and Bing Yao: proposed the study, designed the investigation and revised the manuscript. The final manuscript has been read and approved by all the authors.

Data availability

The data will be made available upon request.

Conflicts of Competing Interest

The authors of this paper affirm that they have no discernible financial conflicts of interest or personal affiliations with any individuals or entities that may have influenced the findings presented herein.

Reference

1. Eisenberg ML, Esteves SC, Lamb DJ, Hotaling JM, Giwercman A, Hwang K, et al. Male infertility. Nat Rev Dis Primers. 2023;9:49.
2. Vander Borght M, Wyns C. Fertility and infertility: Definition and epidemiology. Clinical Biochemistry. 2018;62:2–10.
3. Sharma A, Minhas S, Dhillo WS, Jayasena CN, Sharma A, Minhas S, et al. Male infertility due to testicular disorders. J Clin Endocrinol Metab. 2021;106:e442–59.
4. Olesen IA, Sonne SB, Hoei-Hansen CE, Rajpert-DeMeyts E, Skakkebaek NE. Environment, testicular dysgenesis and carcinoma in situ testis. Best Practice & Research Clinical Endocrinology & Metabolism. 2007;21:462–78.
5. Bashir Q, Thall PF, Milton DR, Fox PS, Kawedia JD, Kebriaei P, et al. Conditioning with busulfan plus melphalan versus melphalan alone before autologous haemopoietic cell transplantation for multiple myeloma: an open-label, randomised, phase 3 trial. The Lancet Haematology. 2019;6:e266–75.
6. Poganitsch-Korhonen M, Masliukaite I, Nurmio M, Lähteenmäki P, van Wely M, van Pelt AMM,

et al. Decreased spermatogonial quantity in prepubertal boys with leukaemia treated with alkylating agents. Leukemia. 2017;31:1460–3.

7. Deng C-H, Liu G-H, Xie Y, Deng C-C, Ouyang B, Lv L-Y, et al. Establishing a nonlethal and efficient mouse model of male gonadotoxicity by intraperitoneal busulfan injection. Asian J Androl. 2020;22:184.

8. Jeyendran RS, Caroppo E, Rouen A, Anderson A, Puscheck E. Selecting the most competent sperm for assisted reproductive technologies. Fertility and Sterility. 2019;111:851–63.

9. Bhang DH, Kim B-J, Kim BG, Schadler K, Baek K-H, Kim YH, et al. Testicular endothelial cells are a critical population in the germline stem cell niche. Nat Commun. 2018;9:4379.

10. Pérez-Crespo M, Pericuesta E, Pérez-Cerezales S, Arenas MI, Lobo MV, Díaz-Gil JJ, et al. Effect of liver growth factor on both testicular regeneration and recovery of spermatogenesis in busulfan-treated mice. Reprod Biol Endocrinol. 2011;9:21.

11. Cui Y, Ren L, Li B, Fang J, Zhai Y, He X, et al. Melatonin Relieves Busulfan-Induced Spermatogonial Stem Cell Apoptosis of Mouse Testis by Inhibiting Endoplasmic Reticulum Stress. Cell Physiol Biochem. 2017;44:2407–21.

12. Jeon YG, Kim YY, Lee G, Kim JB. Physiological and pathological roles of lipogenesis. Nat Metab. 2023;5:735–59.

13. Jensen TK, Priskorn L, Holmboe SA, Nassan FL, Andersson A-M, Dalgård C, et al. Associations of Fish Oil Supplement Use With Testicular Function in Young Men. JAMA Netw Open. 2020;3:e1919462.

14. Jing J, Ouyang L, Zhang H, Liang K, Ma R, Ge X, et al. Omega-3 polyunsaturated fatty acids and its metabolite <scp>12-HEPE</scp> rescue busulfan disrupted spermatogenesis via target to <scp>GPR120</scp>. Cell Proliferation. 2023;n/a:e13551.

15. Collodel G, Moretti E, Noto D, Corsaro R, Signorini C. Oxidation of Polyunsaturated Fatty Acids as a Promising Area of Research in Infertility. Antioxidants. 2022;11:1002.

16. Wang C, Sun C, Lu W, Gul K, Mata A, Fang Y, et al. Emulsion structure design for improving the oxidative stability of polyunsaturated fatty acids. Comp Rev Food Sci Food Safe. 2020;19:2955–71.

17. Nevin KG, Rajamohan T, Nevin KG, Rajamohan T. Beneficial effects of virgin coconut oil on lipid parameters and in vitro LDL oxidation. Clinical Biochemistry. 2004;37:830–5.

18. German JB, Dillard CJ, German JB, Dillard CJ, German JB, Dillard CJ, et al. Saturated Fats: A Perspective from Lactation and Milk Composition. Lipids. 2010;45:915–23.

19. Zhang XS, Zhang P, Liu YH, Xu Q, Zhang Y, Li HZ, et al. Caprylic Acid Improves Lipid Metabolism, Suppresses the Inflammatory Response and Activates the ABCA1/p-JAK2/p-STAT3 Signaling Pathway in C57BL/6J Mice and RAW264.7 Cells. Biomed Environ Sci. 2022;35:95–106.

20. Wang D, Chen J, Sun H, Chen W, Yang X. MCFA alleviate H2 O2 -induced oxidative stress in AML12 cells via the ERK1/2/Nrf2 pathway. Lipids. 2022;57:153–62.

21. He K, Cao C, Xu X, Ye Z, Ma X, Chen W, et al. Octanoic acid–rich enteral nutrition prevented lipopolysaccharide-induced acute liver injury through c-Jun N-terminal kinase–dependent autophagy. J Parenter Enteral Nutr. 2022;46:1353–60.

22. Ge X, He Z, Cao C, Xue T, Jing J, Ma R, et al. Protein palmitoylation-mediated palmitic acid sensing causes blood-testis barrier damage via inducing ER stress. Redox Biology. 2022;54:102380.

23. Zhao J, Wang M, Wang Y, Xu J, Ma C, Tang Y, et al. Endoplasmic reticulum stress promotes blood-testis barrier impairment in mice with busulfan-induced oligospermia through PERK-eIF2α

signaling pathway. Toxicology. 2022;473:153193.

24. Jia X, Xu Y, Wu W, Fan Y, Wang G, Zhang T, et al. Aroclor1254 disrupts the blood–testis barrier by promoting endocytosis and degradation of junction proteins via p38 MAPK pathway. Cell Death Dis. 2017;8:e2823–e2823.

25. Jiang S, Xu Y, Fan Y, Hu Y, Zhang Q, Su W. Busulfan impairs blood–testis barrier and spermatogenesis by increasing noncollagenous 1 domain peptide via matrix metalloproteinase 9. Andrology. 2022;10:377–91.

26. Zhang X, Xue C, Xu Q, Zhang Y, Li H, Li F, et al. Analysis of Relative Gene Expression Data Using Real-Time Quantitative PCR and the 2−ΔΔCT Method. Methods. 2001;25:402–8.

27. Voller B, Lines E, McCrossin G, Tinaz S, Lungu C, Grimes G, et al. Dose-escalation study of octanoic acid in patients with essential tremor. J Clin Invest. 2016;126:1451–7.

28. Cansız D, Ünal İ, Üstündağ ÜV, Alturfan AA, Altinoz MA, Elmacı İ, et al. Caprylic acid ameliorates rotenone induced inflammation and oxidative stress in the gut-brain axis in Zebrafish. Mol Biol Rep. 2021;48:5259–73.

29. Wang D, Chen J, Sun H, Chen W, Yang X. MCFA alleviate H2O2-induced oxidative stress in AML12 cells via the ERK1/2/Nrf2 pathway. Lipids. 2022;57:153–62.

30. Fredrickson J, Krisher R, Morbeck DE. The impact of the protein stabilizer octanoic acid on embryonic development and fetal growth in a murine model. J Assist Reprod Genet. 2015;32:1517–24.

31. Thirouard L, Holota H, Monrose M, Garcia M, De Haze A, Saru J-P, et al. Analysis of the Reversible Impact of the Chemodrug Busulfan on Mouse Testes. Cells. 2021;10:2403.

32. Zhao L, Yao C, Xing X, Jing T, Li P, Zhu Z, et al. Single-cell analysis of developing and azoospermia human testicles reveals central role of Sertoli cells. Nat Commun. 2020;11:5683.

33. Mruk DD, Cheng CY, Mruk DD, Cheng CY, Mruk DD, Cheng CY, et al. The Mammalian Blood-Testis Barrier: Its Biology and Regulation. Endocr Rev. 2015;36:564–91.

34. Chen X, Liang M, Wang D. Progress on the study of the mechanism of busulfan cytotoxicity. Cytotechnology. 2018;70:497–502.

35. Bisht S, Faiq M, Tolahunase M, Dada R. Oxidative stress and male infertility. Nat Rev Urol. 2017;14:470–85.

36. Zhang X, Xia Q, Wei R, Song H, Mi J, Lin Z, et al. Melatonin protects spermatogonia from the stress of chemotherapy and oxidation via eliminating reactive oxidative species. Free Radical Bio Med. 2019;137:74–86.

37. Sürmen MG, Sürmen S, Cansız D, Ünal İ, Üstündağ ÜV, Alturfan AA, et al. Amelioration of rotenone-induced alterations in energy/redox system, stress response and cytoskeleton proteins by octanoic acid in zebrafish: A proteomic study. J Biochem & Molecular Tox. 2022;36:e23024.

38. Mizushima N, Levine B. Autophagy in Human Diseases. Longo DL, editor. N Engl J Med. 2020;383:1564–76.

39. Wang M, Zeng L, Su P, Ma L, Zhang M, Zhang Y zhen. Autophagy: a multifaceted player in the fate of sperm. Genetics in Medicine. 2022;28:200–31.

40. Liu C, Wang H, Shang Y, Liu W, Song Z, Zhao H, et al. Autophagy is required for ectoplasmic specialization assembly in sertoli cells. Autophagy. 2016;12:814–32.

41. Wei R, Zhang X, Cai Y, Liu H, Wang B, Zhao X, et al. Busulfan Suppresses Autophagy in Mouse Spermatogonial Progenitor Cells via mTOR of AKT and p53 Signaling Pathways. Stem Cell Res Ther. 2020;16:1242–55.

42. Yan Q, Zhang Y, Wang Q, Yuan L. Autophagy: A Double-Edged Sword in Male Reproduction. IJMS. 2022;23:15273.
43. Horibe A, Eid N, Ito Y, Otsuki Y, Kondo Y. Ethanol-Induced Autophagy in Sertoli Cells Is Specifically Marked at Androgen-Dependent Stages of the Spermatogenic Cycle: Potential Mechanisms and Implications. IJMS. 2019;20:184.
44. Yang Q, Li F, Miao Y, Luo X, Dai S, Liu J, et al. CdSe/ZnS quantum dots induced spermatogenesis dysfunction via autophagy activation. Journal of Hazardous Materials. 2020;398:122327.
45. Liu S, Huang L, Geng Y, He J, Chen X, Xu H, et al. Rapamycin inhibits spermatogenesis by changing the autophagy status through suppressing mechanistic target of rapamycin-p70S6 kinase in male rats. Mol Med Rep. 2017;16:4029–37.
46. Yi WEI, Xiang-Liang T, Yu Z, Bin L, Lian-Ju S, Chun-lan L, et al. DEHP exposure destroys blood-testis barrier (BTB) integrity of immature testes through excessive ROS-mediated autophagy. Genes & Diseases. 2018;5:263–74.
47. Kadota Y, Toyoda T, Hayashi-Kato M, Kitaura Y, Shimomura Y. Octanoic acid promotes branched-chain amino acid catabolisms via the inhibition of hepatic branched-chain alpha-keto acid dehydrogenase kinase in rats. Metabolism. 2015;64:1157–64.
48. Haynes VR, Michael NJ, van den Top M, Zhao F-Y, Brown RD, De Souza D, et al. A Neural basis for Octanoic acid regulation of energy balance. Molecular Metabolism. 2020;34:54–71.
49. Carnielli VP, Sulkers EJ, Moretti C, Wattimena JLD, van Goudoever JB, Degenhart HJ, et al. Conversion of octanoic acid into long-chain saturated fatty acids in premature infants fed a formula containing medium-chain triglycerides. Metabolism. 1994;43:1287–92.
50. Jain S, Rai R, Singh D, Vohora D, Jain S, Rai R, et al. Octanoic acid a major component of widely consumed medium-chain triglyceride ketogenic diet is detrimental to bone. Sci Rep. 2021;11:7003.

Figure legends

Fig. 1 Analysis of fatty acid and INHB levels in the serum and sperm. (A) Heatmap of the serum fatty acid composition. Control, healthy individuals (n=30); AZO, patients with azoospermia (n=30) AZO26, 28 and 30 represent EO, while the others represent NOA. (B-C) Fatty acid levels of C6 (B) and C8 (C) in serum. (D) Sperm concentration. (E) Serum INHB levels. INHB, inhibin B. The data are presented as the mean ± SD. Statistical analyses were carried out using two-tailed Student's t tests (****$P < 0.0001$). EO, extreme oligospermia; NOA, nonobstructive azoospermia.

Fig. 2 Experimental design and the effects of OCA and busulfan on spermatogenesis. (A) Experimental design. All animals were randomly divided into a control group, a busulfan group and four treatment groups with OCA (32, 64, 128, and 256 mg/kg BW every two days). (B-E) Sperm concentration (B), sperm PR (C), organ coefficient of the testis (D) and epididymis (E) of the mice

at 9 weeks after OCA treatment (n=7~10). (F) Representative photographs of testicular morphology with HE staining. Scale bars: 100 μm and 20 μm. The black asterisk (*) stand for damaged spermatogenic tubules. SPG, spermatogonia; SPM, spermatocyte; SP, spermatozoa; SC, Sertoli cell. (G-H) The width of the seminiferous epithelium (G) and diameter of the seminiferous tubules (H) were calculated randomly from 50 cross-sections of round or nearly round seminiferous tubules (long axis: short axis < 1.2:1). (I) Proportion of different seminiferous tubules calculated from 10 random fields for each group. The data are presented as the mean ± SD. Statistical analyses were carried out using one-way ANOVA followed by Tukey's post hoc test. $^{****}P < 0.0001$ vs. the control group, $^{\#\#\#\#}P < 0.0001$ vs. the busulfan group.

Fig. 3 The effects of OCA and busulfan on the blood-testes barrier in mice. (A) Representative fluorescence images of BTB integrity detected by the biotin tracer assay. The white asterisk (*) indicates the permeation of biotin into the seminiferous lumen. Scale bars: 100 μm. (B) Box plot illustrating the extent of BTB damage, which was calculated randomly from 50-60 seminiferous tubules for each group. (C-D) Representative images of tight junction proteins were examined by western blotting in mouse testes (n=3). (E-H) The relative protein expression of ZO-1 (E), Occludin (F), Claudin5 (G) and Claudin11 (H) was quantified by ImageJ and normalized to vinculin or β-actin levels (n=3). The data are presented as the mean ± SD. Statistical analyses were carried out using one-way ANOVA followed by Tukey's post hoc test. $^{*}P < 0.05$, $^{**}P < 0.01$, $^{****}P < 0.0001$ vs. Control group, $^{\#}P < 0.05$, $^{\#\#}P < 0.01$, $^{\#\#\#\#}P < 0.0001$ vs. Busulfan group. ZO-1, zona occluden-1.

Fig. 4 Changes in oxidative stress levels in the testis after busulfan injection with or without OCA supplementation. (A-B) SOD activity and MDA levels in the testes of mice (n=6~8). (C-F) The mRNA levels of NQO1 (C), CAT (D), SOD3 (E) and HO1 (F) were examined by qPCR and normalized to β-actin levels. (n=4~6). (G) Representative images of HO1 and NQO1 protein expression in testes were examined by western blotting. (H-I) The protein expression levels of HO1 (H) and NQO1 (I) were quantified by ImageJ and normalized to β-actin levels (n=3). (J) Representative immunofluorescence images of P62 (red) and LC3B (red) in the testis. Scale bar:

100 μm. (K) Representative images of LC3B and P62 protein expression in testes were examined by western blotting (n=3). (L-M) The protein expression levels of P62 (L) and LC3B (M) were quantified by ImageJ and normalized to β-actin levels (n=3). The data are presented as the mean ± SD. Statistical analyses were carried out using one-way ANOVA followed by Tukey's post hoc test. *P < 0.05, ***P < 0.001 vs. Control group, $^{\#}P < 0.05$, $^{\#\#\#}P < 0.001$ vs. Busulfan group. SOD: superoxide dismutase; CAT, catalase; NQO1: NAD(P)H, quinone oxidoreductase 1; HO1, heme oxygenase 1; P62, SQSTM1, sequestosome 1; LC3, light chain 3.

Fig. 5 Effects of OCA and busulfan on oxidative stress in TM4 cells. (A) Analysis of the cell survival-rescuing effects of OCA after busulfan treatment by CCK-8 (n = 5). (B) TER detection of TM4 cell barriers (n = 4). (C-D) Representative images of ZO-1, Occludin, HO1, NQO1, LC3B and P62 proteins were examined by western blotting. (E) The relative protein expression levels were quantified by ImageJ and normalized to β-actin or Vinculin levels (n=3). (F) Representative images of ROS generation in the busulfan group and OCA pretreatment group. Scale bar: 100 μm. (G) Representative images of the formation of autophagosomes by GFP-LC3 immunofluorescence; scale bar = 10 μm. Quantification of the number of GFP-positive puncta in each TM4 cell line (n=4). The data are presented as the means ± SDs. Statistical analyses were carried out using one-way ANOVA followed by Tukey's post hoc test. *P < 0.05, **P < 0.01, ***P < 0.001, ****P < 0.0001 vs. the control group; $^{\#}P < 0.05$, $^{\#\#}P < 0.01$ vs. the busulfan group. ZO-1, zona occluden-1; NQO1: NAD(P)H, quinone oxidoreductase 1; HO1, heme oxygenase 1; P62, SQSTM1, sequestosome 1; LC3, light chain 3.

Fig. 6 The effect of rapamycin treatment with or without OCA supplementation on autophagy in TM4 cells. (A) Analysis of cell viability after treatment with rapamycin (0, 0.1, 0.5, 1, 2, 5, 10, or 20 μM) for 24 h (n = 6). (B) Analysis of the cell survival-rescuing effects of OCA (100, 200, or 400 μM) after rapamycin treatment by CCK-8 (n = 5). (C) TER detection of TM4 cell barriers (n = 4). (D) Representative images of ZO-1 and Occludin proteins were examined by western blotting. (E) The relative protein expression levels were quantified by ImageJ and normalized to Vinculin levels (n=3). (F) Representative images of the formation of autophagosomes by GFP-LC3

immunofluorescence; scale bar = 10 μm. (G) Quantification of the number of GFP-positive puncta in each TM4 cell line; n=4. (H) Representative images of the LC3B and P62 proteins were examined by western blotting. (I) The relative protein expression levels were quantified by ImageJ and normalized to the β-actin levels (n=3). For A and B, statistical analyses were carried out using one-way ANOVA followed by Dunnett's multiple comparisons test. For C-F, statistical analyses were carried out using one-way ANOVA followed by Tukey's post hoc test. The data are presented as the mean ± SD. $^{*}P < 0.05$, $^{**}P < 0.01$, $^{****}P < 0.0001$ vs. Control group, $^{\#}P < 0.05$, $^{\#\#\#}P < 0.001$, vs. Busulfan group. P62, SQSTM1, sequestosome 1; LC3, light chain 3; ZO-1, zona occluden-1.

Fig. 7 Mechanistic diagram of OCA and busulfan regulating Sertoli cell barrier function. Busulfan induces oxidative stress and autophagy in Sertoli cells, leading to downregulation of tight junction protein expression and ultimately disruption of the Sertoli cell barrier. Conversely, OCA alleviated oxidative stress and autophagy to ameliorate Sertoli cell dysfunction induced by busulfan. OCA, octanoic acid; ROS, reactive oxygen species; ZO-1, zona occluden-1; LC3II, light chain 3 II.

Fig. S1 The effect of OCA and BUS on spermatogenesis in mice. (A-B) testis weight (A) and epididymis weight (B) of the mice at 9 weeks after OCA treatment (n=7~10). (C) Representative photographs of epididymal cauda with HE staining. Asterisk (＊) indicated decrease of sperm density in tubules of epididymal cauda. Scale bars: 100 μm. Date are presented as mean ± SD. Statistical analyses were carried out using one-way ANOVA followed by Tukey's post hoc test. $^{*}P < 0.05$, $^{**}P < 0.01$, $^{****}P < 0.0001$ vs. Control group, $^{\#}P < 0.05$, $^{\#\#}P < 0.01$, $^{\#\#\#}P < 0.001$, $^{\#\#\#\#}P < 0.0001$ vs. Busulfan group.
