# Supplementary material for: Octanoic acid mitigates busulfan-induced blood-testis barrier damage by alleviating oxidative stress and autophagy
Source: Lipids Health Dis. 2024 Jun 11;23:180. doi: 10.1186/s12944-024-02157-2 (PMC11165768; doi:10.1186/s12944-024-02157-2)

Full unedited gel for Figure 3C

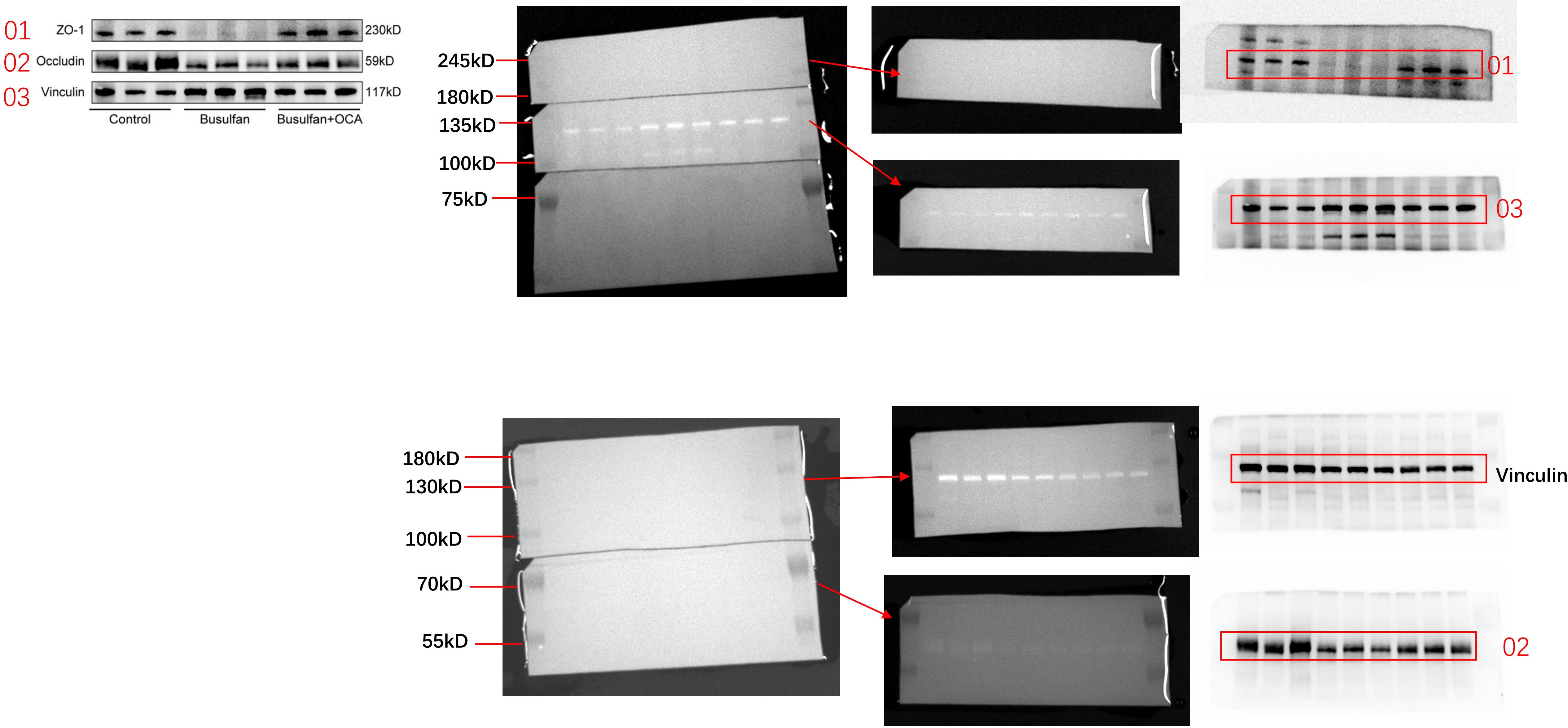

Full unedited gel for Figure 3D

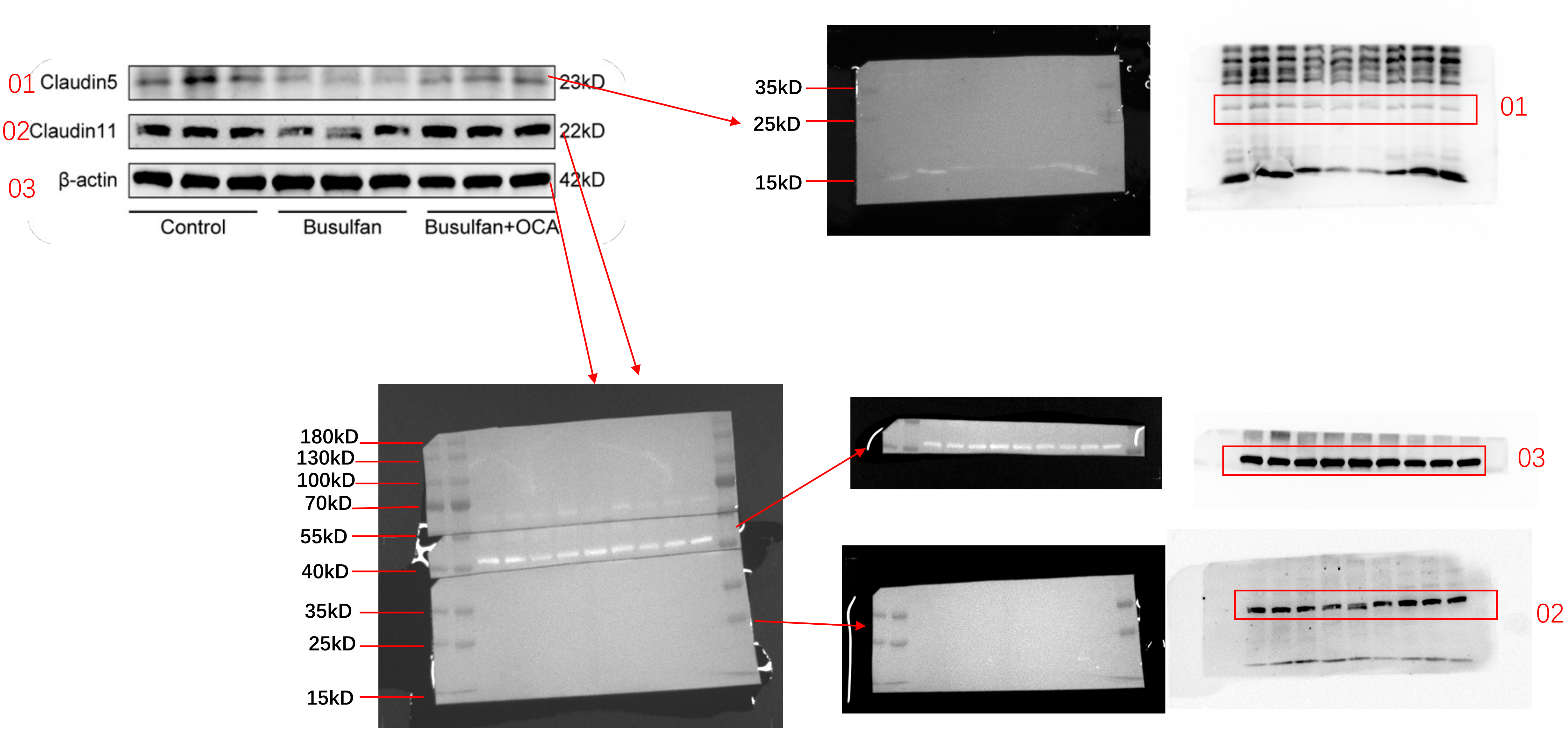

Full unedited gel for Figure 4G

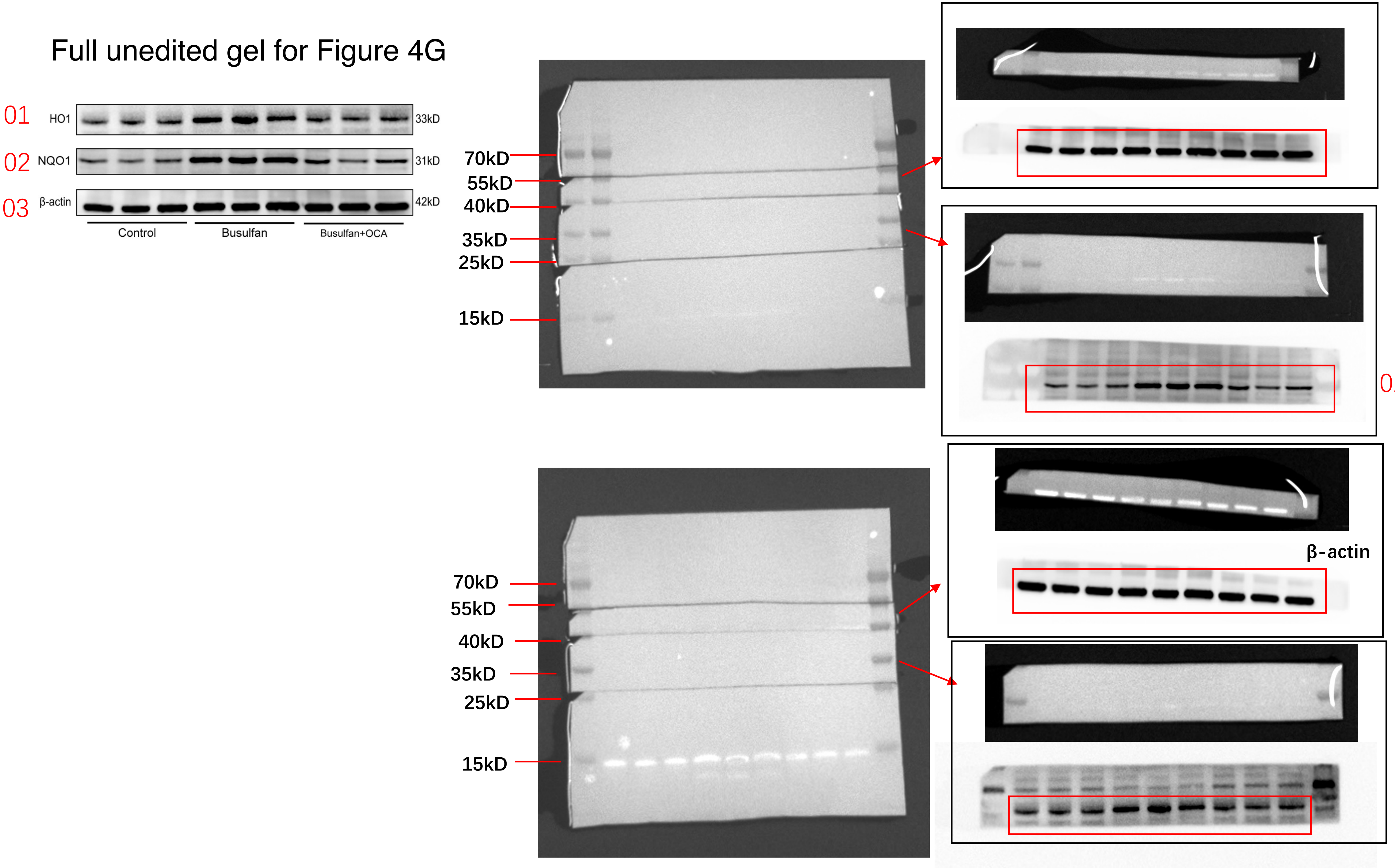

Full unedited gel for Figure 4K

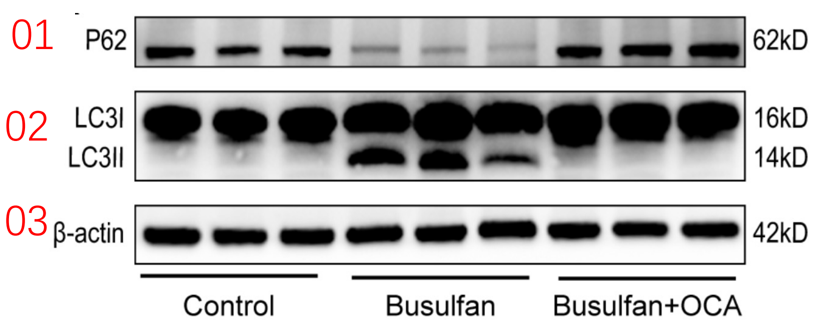

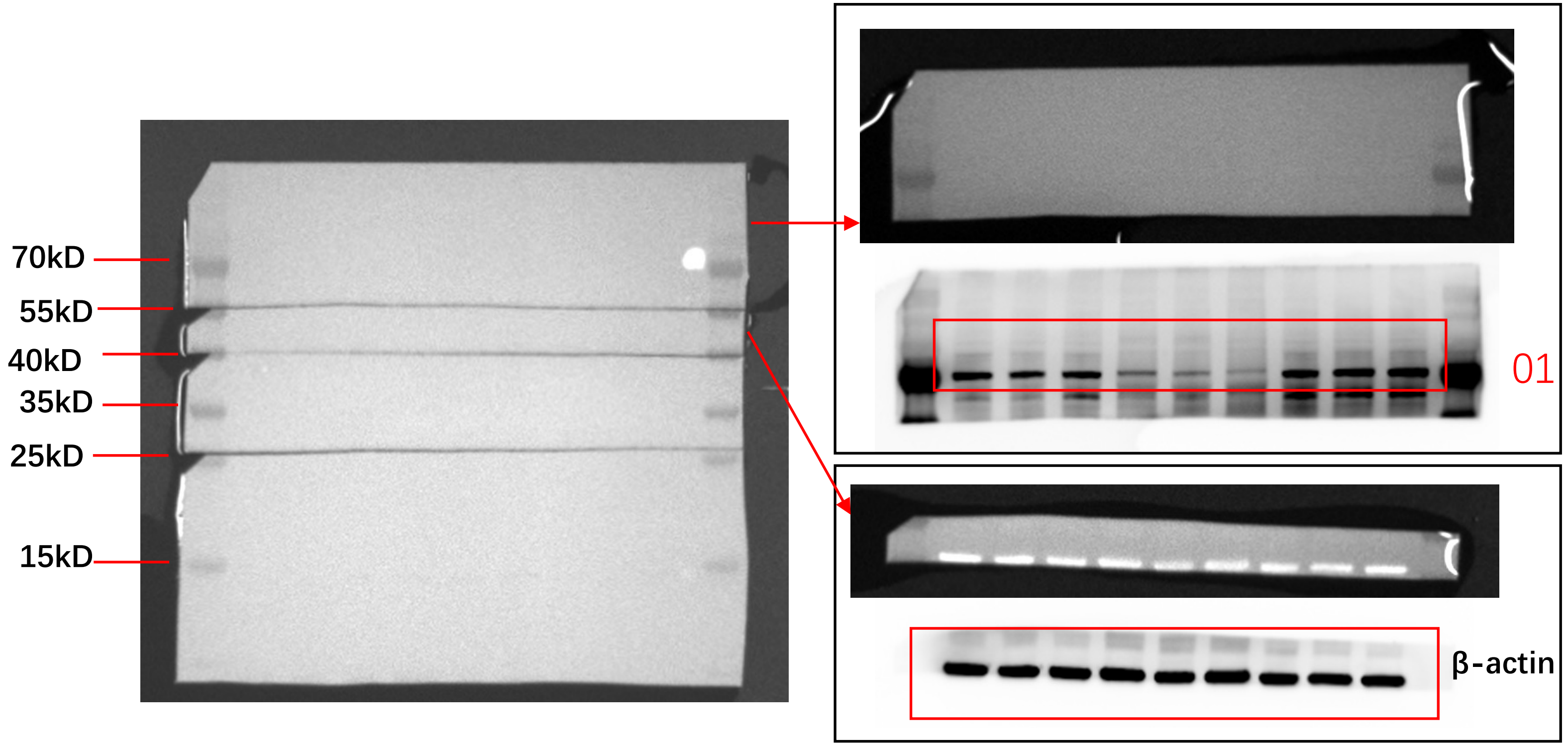

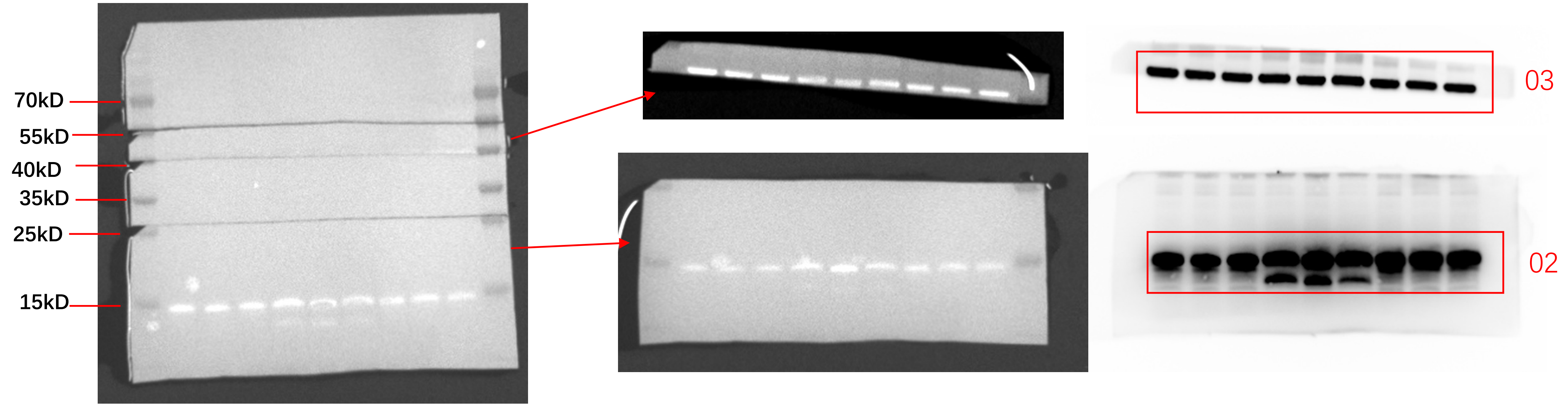

Full unedited gel for Figure 5C

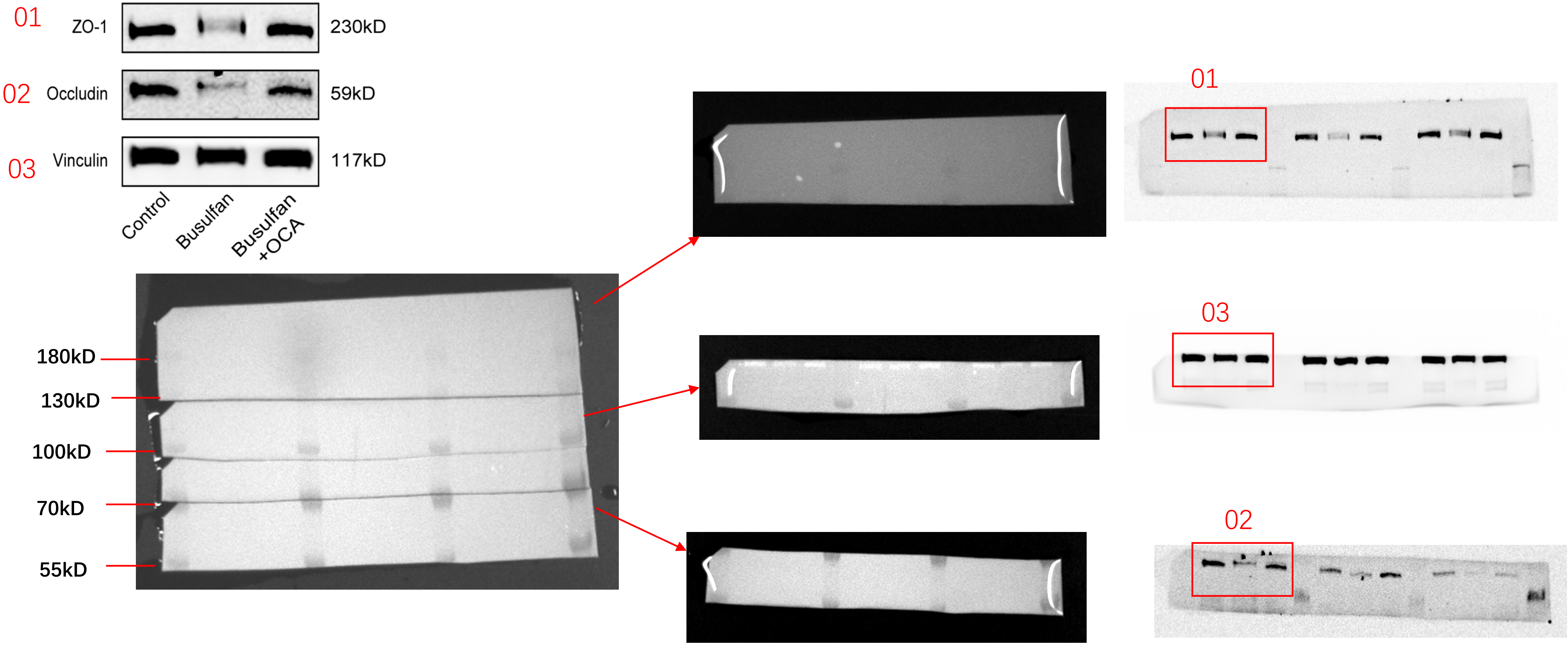

Full unedited gel for Figure 5D

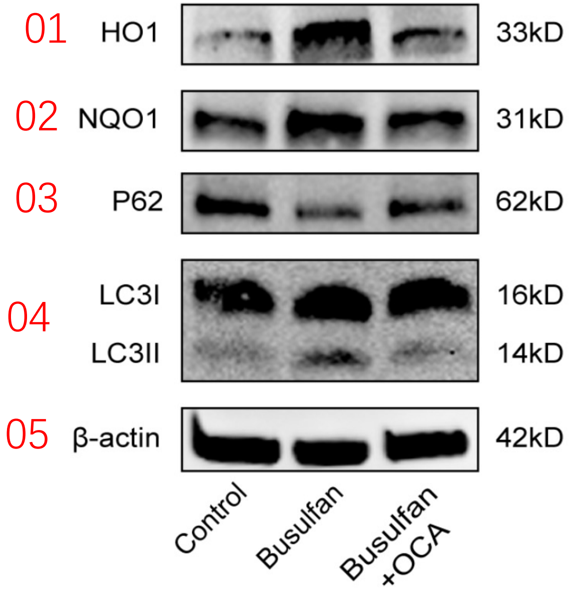

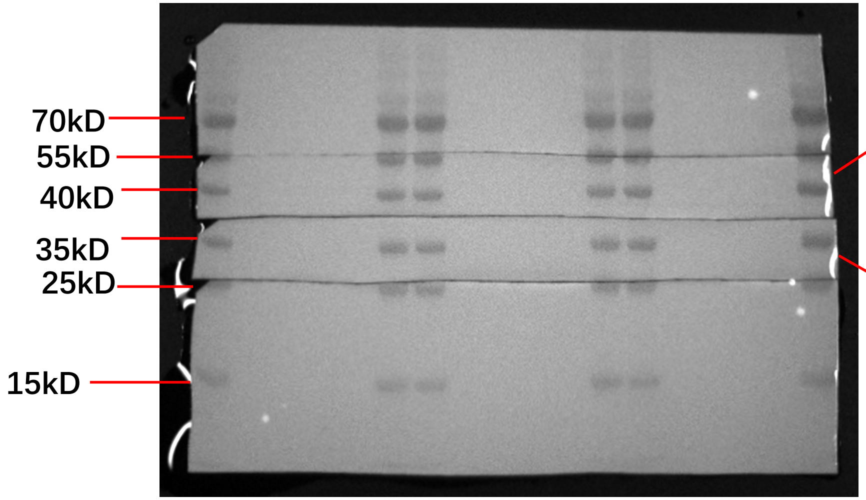

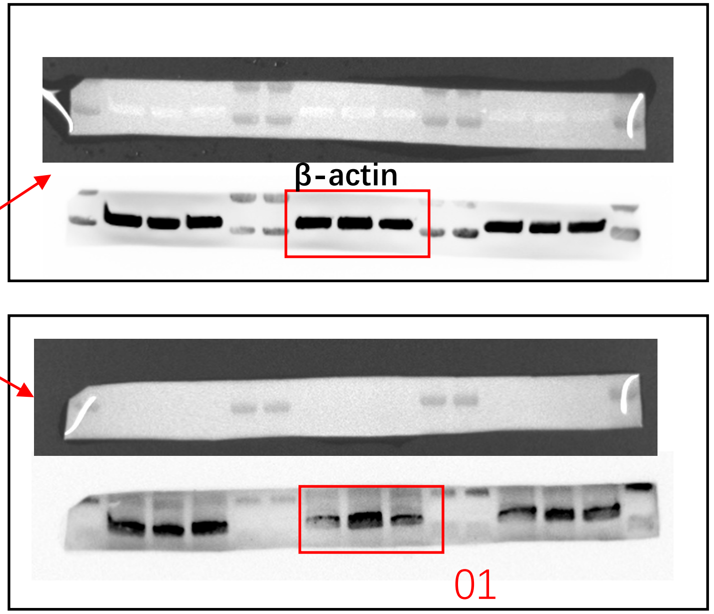

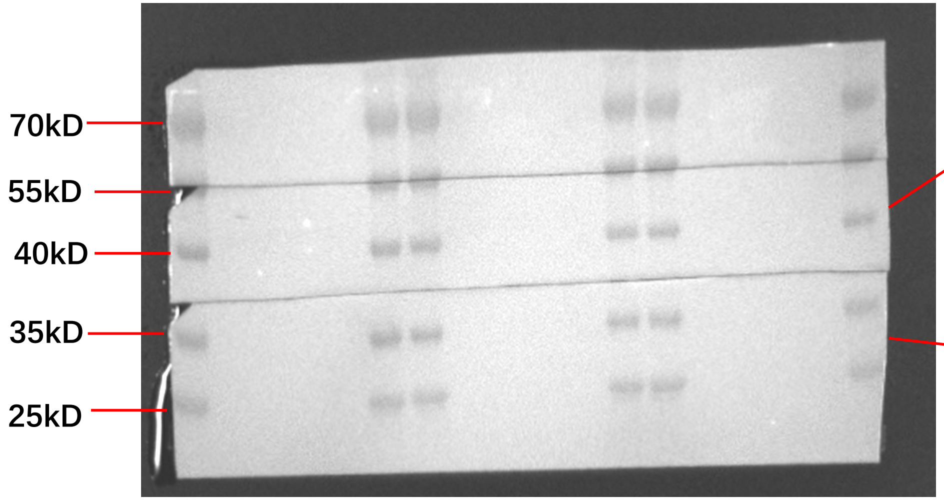

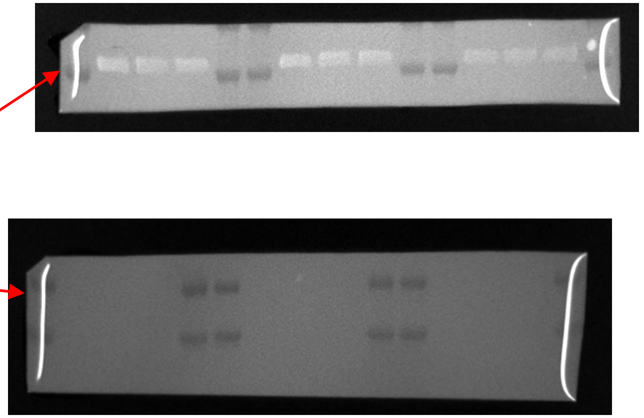

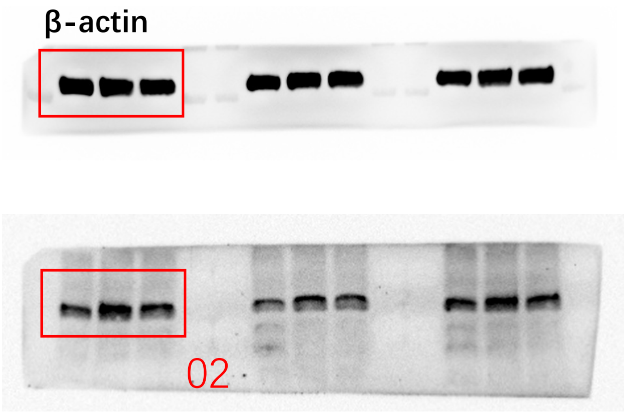

Full unedited gel for Figure 5D

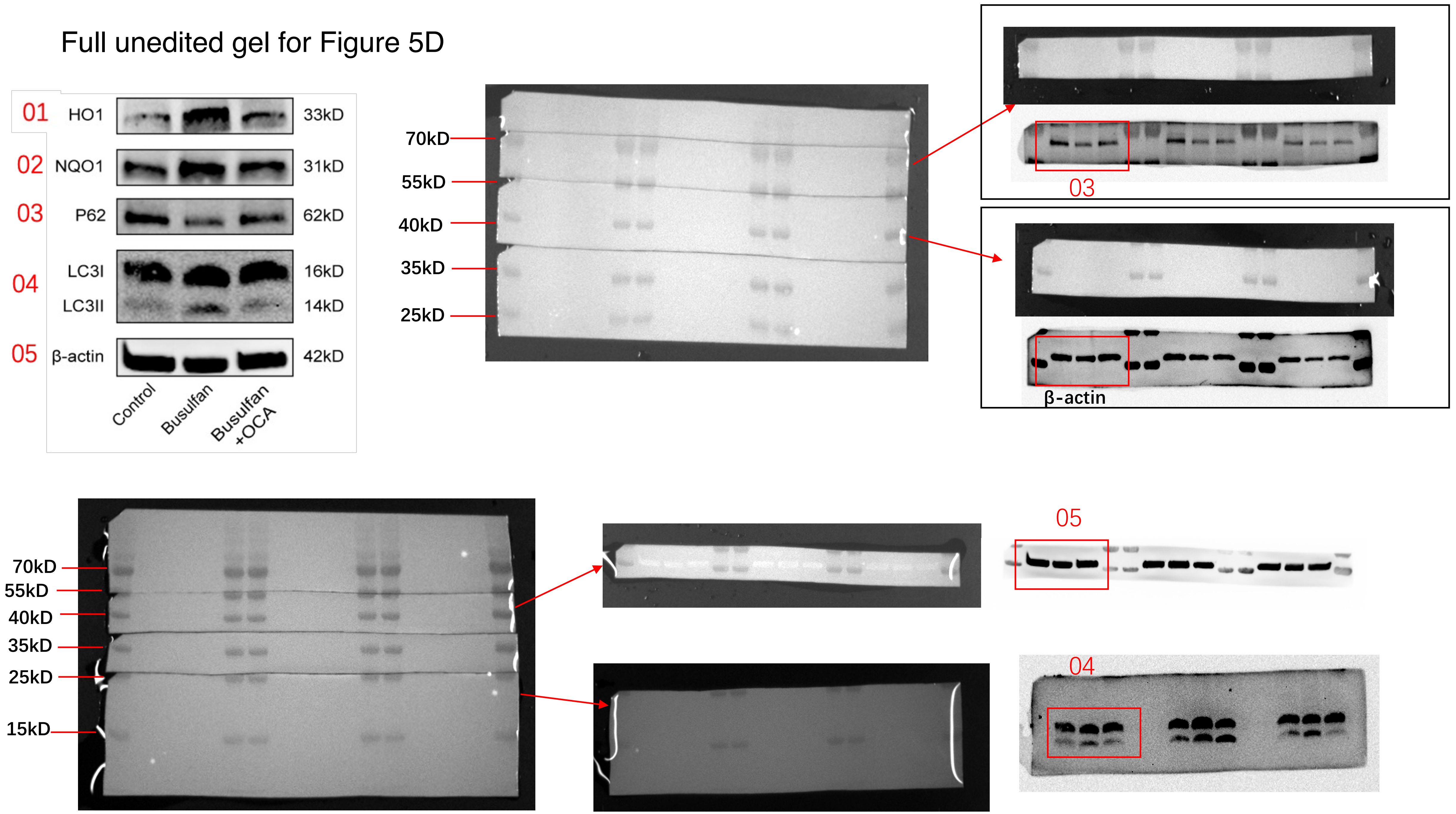

Full unedited gel for Figure 6D

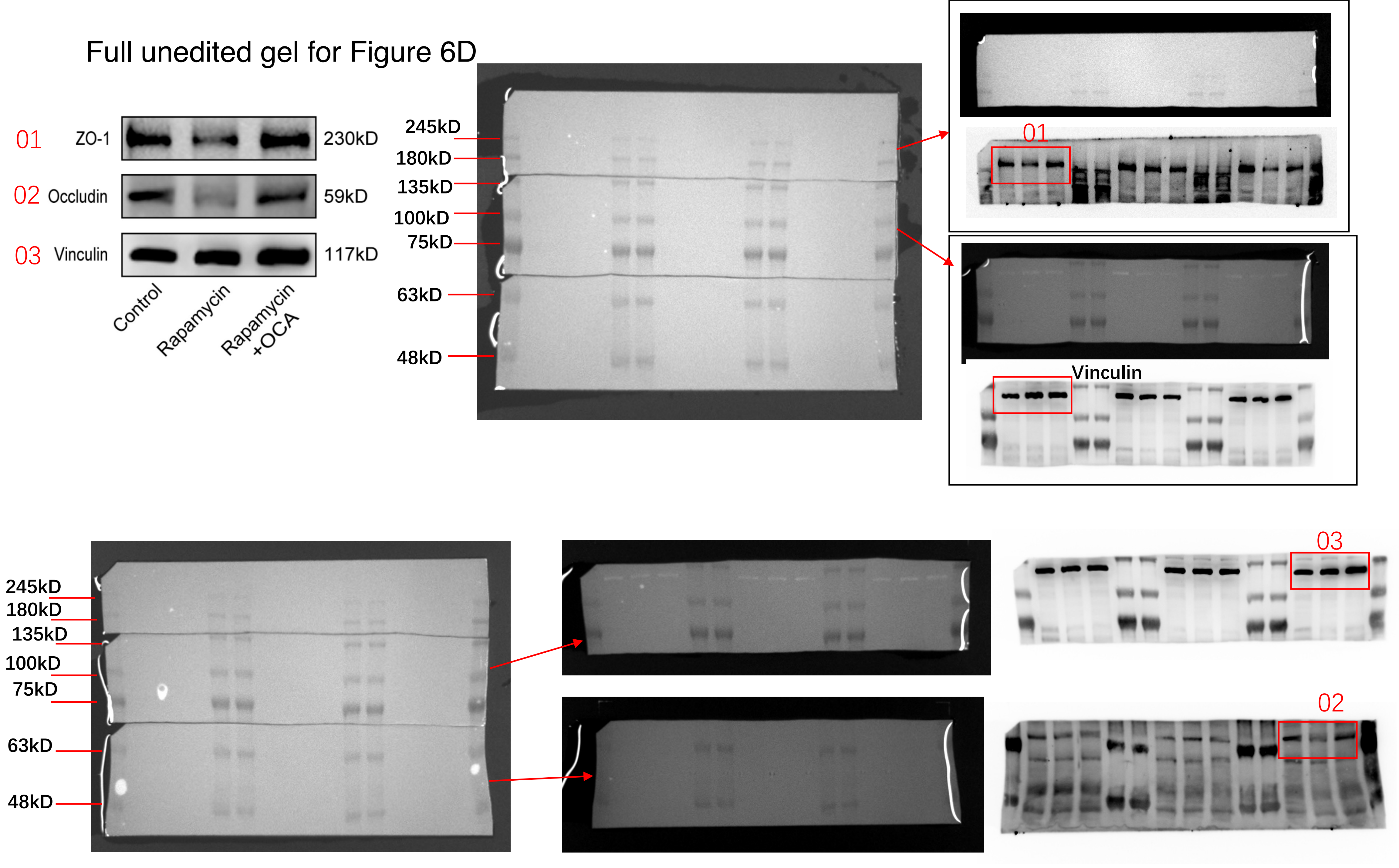

Full unedited gel for Figure 6H

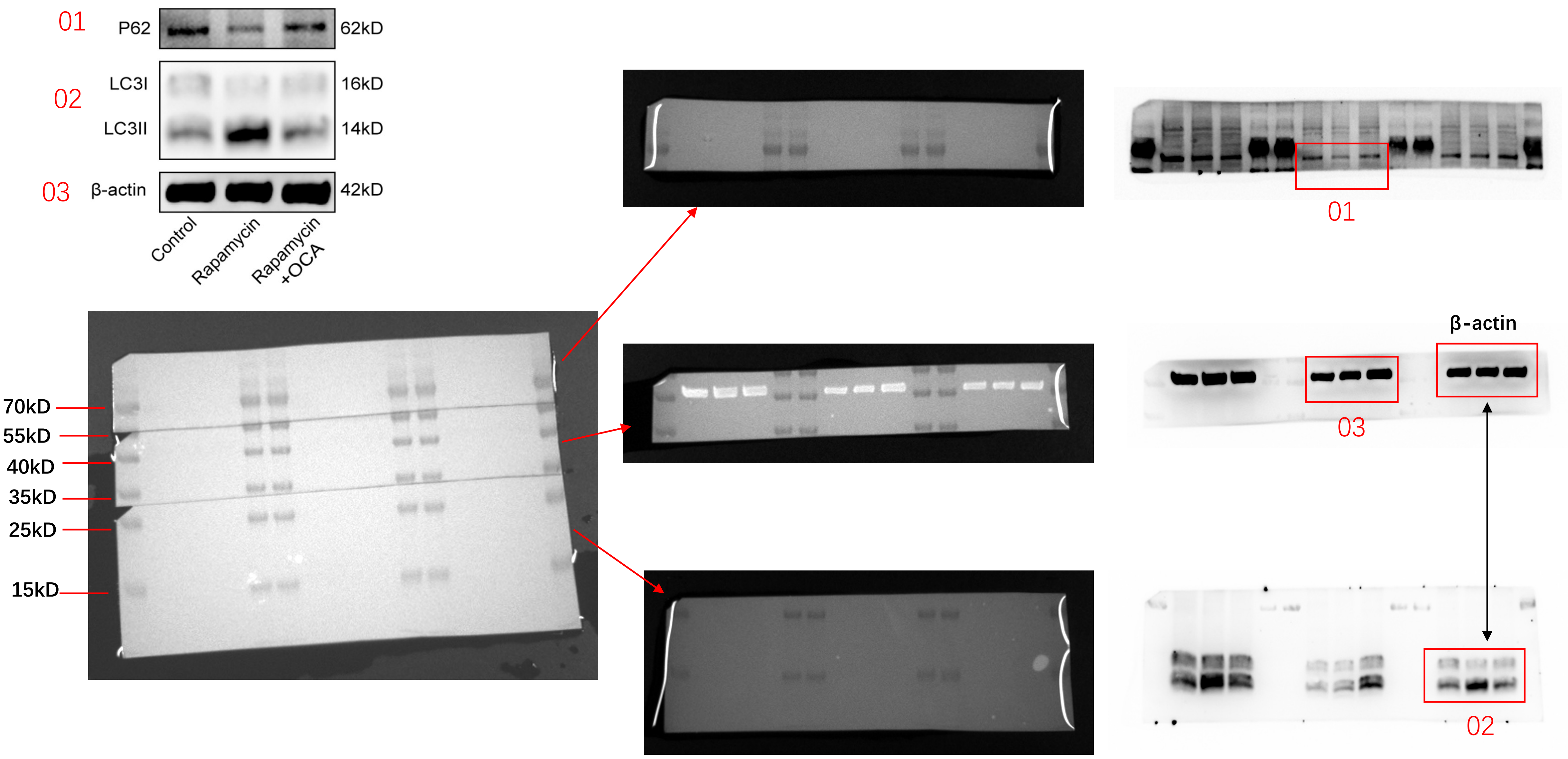

Supplement: Supplementary file 2 — Supplementary Material 2 [file 12944_2024_2157_MOESM2_ESM.pdf]
