# Supplementary material for: Octanoic acid mitigates busulfan-induced blood-testis barrier damage by alleviating oxidative stress and autophagy
Source: Lipids Health Dis. 2024 Jun 11;23:180. doi: 10.1186/s12944-024-02157-2 (PMC11165768; doi:10.1186/s12944-024-02157-2)
Supplement: Supplementary file 3 — Supplementary Material 3 [file 12944_2024_2157_MOESM3_ESM.docx]

**Supplementary Tables**

**Table S1. Summary of the clinical parameters of all study participants.**

| **No.** | **Age** | **Sperm concentration (10^6/mL)** | **Inhibin B (pg/mL)** | **Type** |
| --- | --- | --- | --- | --- |
| 1 | 23 | 99.75 | 180 | Normal |
| 2 | 35 | 51.88 | 142 | Normal |
| 3 | 28 | 67.95 | 129 | Normal |
| 4 | 28 | 148.79 | 130 | Normal |
| 5 | 33 | 30.58 | 103 | Normal |
| 6 | 31 | 71.31 | 113 | Normal |
| 7 | 33 | 26 | 133 | Normal |
| 8 | 29 | 22.02 | 97.8 | Normal |
| 9 | 25 | 75.49 | 130 | Normal |
| 10 | 32 | 80.83 | 196 | Normal |
| 11 | 31 | 69.63 | 127 | Normal |
| 12 | 25 | 87.38 | 169 | Normal |
| 13 | 29 | 105.61 | 117 | Normal |
| 14 | 32 | 60.08 | 209 | Normal |
| 15 | 27 | 79.83 | 178 | Normal |
| 16 | 31 | 36.32 | 126 | Normal |
| 17 | 32 | 43.97 | 137 | Normal |
| 18 | 36 | 45.4 | 135 | Normal |
| 19 | 27 | 40.84 | 95.2 | Normal |
| 20 | 28 | 28.01 | 104 | Normal |
| 21 | 31 | 81.35 | 145 | Normal |
| 22 | 29 | 45.53 | 94.2 | Normal |
| 23 | 26 | 94.74 | 113 | Normal |
| 24 | 31 | 39.16 | 98.5 | Normal |
| 25 | 32 | 94.06 | 162 | Normal |
| 26 | 29 | 44.85 | 93.3 | Normal |
| 27 | 29 | 35.15 | 189 | Normal |
| 28 | 29 | 46.7 | 153 | Normal |
| 29 | 30 | 40.09 | 175 | Normal |
| 30 | 32 | 60.76 | 115 | Normal |
| 31 | 28 | 0.5 | 30.40 | EO |
| 32 | 33 | 3.02 | 35.40 | EO |
| 33 | 32 | 0.33 | 18.79 | NOA |
| 34 | 26 | 0.33 | 39.20 | NOA |
| 35 | 32 | 0.11 | 20.50 | NOA |
| 36 | 30 | 0.34 | 21.30 | NOA |
| 37 | 26 | 0 | 5.38 | NOA |
| 38 | 30 | 0 | 8.34 | NOA |
| 39 | 25 | 0 | 13.30 | NOA |
| 40 | 27 | 0 | 10.90 | NOA |
| 41 | 34 | 0 | 11.20 | NOA |
| 42 | 20 | 0 | 12.00 | NOA |
| 43 | 33 | 0 | 12.80 | NOA |
| 44 | 26 | 0 | 12.30 | NOA |
| 45 | 27 | 0 | 12.50 | NOA |
| 46 | 22 | 0 | 12.90 | NOA |
| 47 | 21 | 0 | 14.10 | NOA |
| 48 | 43 | 0 | 14.60 | NOA |
| 49 | 31 | 0 | 15.50 | NOA |
| 50 | 34 | 0 | 16.10 | NOA |
| 51 | 30 | 0 | 16.70 | NOA |
| 52 | 25 | 0 | 11.50 | NOA |
| 53 | 28 | 0 | 19.90 | NOA |
| 54 | 31 | 0 | 20.20 | NOA |
| 55 | 29 | 0 | 21.30 | NOA |
| 56 | 25 | 0 | 21.40 | NOA |
| 57 | 27 | 0 | 22.40 | NOA |
| 58 | 28 | 0 | 29.10 | NOA |
| 59 | 35 | 0 | 31.80 | NOA |
| 60 | 24 | 0 | 52.60 | EO |

**Table S2. Basic characteristics and parameters of all study participants.**

| Parameters | Control (n=30) | NOA/EO (n=30) | *P* value |
| --- | --- | --- | --- |
| Age (years) |  |  |  |
| Mean (SD) | 30 (2.9) | 29 (4.7) | 0.3147 |
| Range | 23-36 | 20-43 |  |
| Sperm concentration (10^6^/mL) |  |  |  |
| Mean (SD) | 61.802 (28.511) | 0.154 (0.548) | <0.0001 |
| Range | 22.02-148.79 | 0-3.02 |  |
| Inhibin B (pg/mL) |  |  |  |
| Mean (SD) | 136.3 (32.411) | 19.48 (10.038) | <0.0001 |
| Range | 93.3-209 | 5.38-52.6 |  |

**Table S3. Sequences of the primers.**

| **Gene Name** | **Direction** | **Sequence (5’-3’)** |
| --- | --- | --- |
| β-actin (internal control) | F | AGCCATGTACGTAGCCATCC |
|  | R | CTCTCAGCTGTGGTGGTGAA |
| SOD3 | F | CCTTCTTGTTCTACGGCTTGC |
|  | R | TCGCCTATCTTCTCAACCAGG |
| HO-1 | F | CCTCACAGATGGCGTCACTT |
|  | R | TGGGGGCCAGTATTGCATTT |
| NQO1 | F | AGGATGGGAGGTACTCGAATC |
|  | R | AGGCGTCCTTCCTTATATGCTA |
| CAT | F | CCCCTATTGCCGTTCGATTCT |
|  | R | TTCAGGTGAGTCTGTGGGTTT |
