# Supplementary material for: Octanoic acid mitigates busulfan-induced blood-testis barrier damage by alleviating oxidative stress and autophagy
Source: Lipids Health Dis. 2024 Jun 11;23:180. doi: 10.1186/s12944-024-02157-2 (PMC11165768; doi:10.1186/s12944-024-02157-2)
Supplement: Supplementary file 4 — Supplementary Material 4 [file 12944_2024_2157_MOESM4_ESM.pdf]

Editing Certificate

This document certifies that the manuscript

Octanoic acid mitigates busulfan-induced blood-testis barrier damage by alleviating oxidative stress and autophagy

prepared by the authors

Chun Cao, Hong Zhang, Zhaowanyue He, Kemei Zhang, Zhang Qian, Jiaming Shen, Lu Zheng, Mengqi Xue, Shanshan Sun, Chuwei Li, Wei Zhao, Jun Jing, Rujun Ma, Xie Ge, Bing Yao

was edited for proper English language, grammar, punctuation, spelling, and overall style by one or more of the highly qualified native English speaking editors at AJE.

This certificate was issued on **May 9, 2024** and may be verified on the AJE website using the verification code **6158-11A2-E8A1-131C-379P**.

Neither the research content nor the authors' intentions were altered in any way during the editing process. Documents receiving this certification should be English-ready for publication; however, the author has the ability to accept or reject our suggestions and changes. To verify the final AJE edited version, please visit our verification page at aje.com/certificate. If you have any questions or concerns about this edited document, please contact AJE at support@aje.com.

AJE provides a range of editing, translation, and manuscript services for researchers and publishers around the world. For more information about our company, services, and partner discounts, please visit aje.com.
