# Supplementary material for: Octanoic acid mitigates busulfan-induced blood-testis barrier damage by alleviating oxidative stress and autophagy
Source: Lipids Health Dis. 2024 Jun 11;23:180. doi: 10.1186/s12944-024-02157-2 (PMC11165768; doi:10.1186/s12944-024-02157-2)
Supplement: Supplementary file 5 — Supplementary Material 5 [file 12944_2024_2157_MOESM5_ESM.docx]

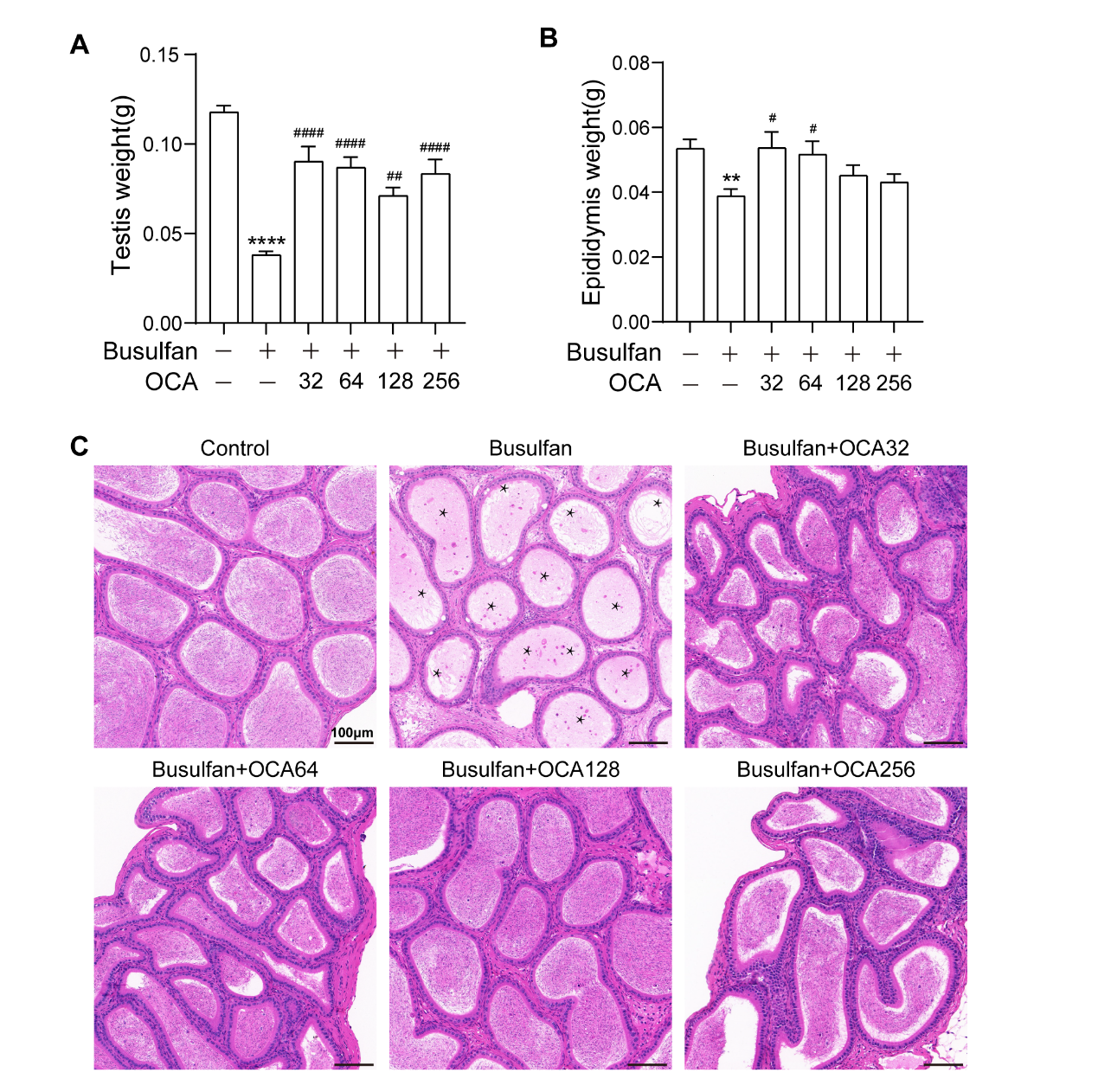


**Fig. S1** **Effect of OCA and busulfan on spermatogenesis in mice.** (A-B) Testis weight (A) and epididymis weight (B) of the mice at 9 weeks after OCA treatment (n=7~10). (C) Representative images of the epididymal cauda with HE staining. Asterisks (＊) indicate a decrease in sperm density in the tubules of the epididymal cauda. Scale bars: 100 μm. The data are presented as the mean ± SD. Statistical analyses were carried out using one-way ANOVA followed by Tukey’s post hoc test. **P* < 0.05, ***P* < 0.01, *****P* < 0.0001 vs. the control group; ^#^*P* < 0.05, ^##^*P* < 0.01, ^###^*P* < 0.001, ^####^*P* < 0.0001 vs. the busulfan group.
